# Prevalence, Trajectory, and Factors Associated With Patient-Reported Nonmotor Outcomes After Stroke: A Systematic Review and Meta-Analysis

**DOI:** 10.1001/jamanetworkopen.2024.57447

**Published:** 2025-02-21

**Authors:** Hatice Ozkan, Gareth Ambler, Taniya Esmail, Gargi Banerjee, Robert J. Simister, David J. Werring

**Affiliations:** 1 Stroke Research Centre, Department of Brain Repair and Rehabilitation, UCL Queen Square Institute of Neurology, University College London, London, United Kingdom; 2 Comprehensive Stroke Service, National Hospital for Neurology and Neurosurgery, University College London Hospitals NHS Foundation, London, United Kingdom; 3 Department of Statistical Science, University College London, London, United Kingdom

## Abstract

**Importance:**

The evidence on nonmotor outcomes after acute ischemic stroke and intracerebral hemorrhage (ICH) is limited and domain-specific. The prevalence, natural history, and factors associated with nonmotor outcomes across multiple domains are unclear.

**Objective:**

To synthesize the published data and to identify the prevalence, natural history, and factors associated with multidomain nonmotor outcome burden in patients with stroke.

**Data Sources:**

A search of PubMed, MEDLINE, EMBASE, and PsycINFO databases between January 1999 to June 2023 was supplemented by search of bibliographies of the key articles.

**Study Selection:**

The analysis included prospective cohort studies that reported nonmotor outcomes across 10 domains: anxiety, depression, fatigue, sleep disturbance, social participation, pain, bladder dysfunction, bowel dysfunction (constipation and fecal incontinence), and sexual dysfunction assessed by patient-reported scales.

**Data Extraction and Synthesis:**

Two reviewers independently assessed studies, extracting baseline data, nonmotor prevalence, and follow-up information. Pooled prevalence of nonmotor outcomes was estimated using random-effects models. Meta-regression models were used to analyze natural history and factors associated with nonmotor outcomes. Subgroup analysis was used to assess prevalence by symptom description within each nonmotor domain. Publication bias and study quality were assessed using funnel plots and the Newcastle-Ottawa scale.

**Results:**

A total of 279 prospective cohort studies met the inclusion criteria (117 440 participants with stroke; median [IQR] age, 65 [59-70] years; 209 of 279 studies with more male than female participants) with a nonmotor outcome follow-up period ranging from 30 days to 10 years after stroke. The most prevalent adverse nonmotor outcomes by pooled prevalence were sleep disturbance (59.9%; 95% CI, 53.9%-63.9%), sexual dysfunction (59.8%; 95% CI, 50.0%-69.5%), constipation (58.2%; 95% CI, 53.9%-62.6%), reduced social participation (56.5%; 95% CI, 52.1%-60.8%), bladder dysfunction (45.9%; 95% CI, 38.0%-53.8%), and fatigue (45.2%; 95% CI, 40.7%-49.5%). Meta-regression analysis showed no significant improvement over time for most nonmotor outcomes, except pain (coefficient = −11.0%; *P* = .05) and sexual dysfunction (coefficient = −24.1%; *P* < .001). The heterogeneity ranged between 52% and 98% across all studies. The common factors associated with adverse nonmotor outcomes were female sex, studies with mixed stroke cohort (ischemic stroke or ICH), and older age.

**Conclusions and Relevance:**

Patient-reported nonmotor outcomes were common after stroke. Sexual dysfunction, sleep disturbance, constipation, reduced social participation, bladder dysfunction, and fatigue were most prevalent. These adverse outcomes often persisted over time, especially in women, older adults, and those in studies with mixed stroke cohorts.

## Key Points

**Question:**

What are the prevalence, natural history, and factors associated with adverse nonmotor outcomes in multiple health-related domains after stroke?

**Findings:**

This systematic review and meta-analysis, including 279 studies with 117 440 patients, found a high prevalence of adverse nonmotor outcomes across 10 domains, with sleep disturbance, sexual dysfunction, constipation, reduced social participation, bladder dysfunction, and fatigue being the most prevalent. Most domains increased in prevalence or were unchanged over time, and significant factors associated with the prevalence of nonmotor outcomes included female sex, mixed stroke cohort, and older age.

**Meaning:**

These findings suggest that nonmotor outcomes are prevalent after stroke, highlighting the need for multifaceted approaches for long-term management.

## Teaser

This systematic review and meta-analysis evaluates the prevalence, natural history, and factors associated with multidomain nonmotor outcome burden in patients with stroke.

## Introduction

Stroke outcomes are commonly assessed using the modified Rankin Score, which focuses on motor function and walking ability but neglects nonmotor outcomes.^1^ This approach limits comprehensive understanding of recovery and quality of life after stroke.^2^ Nonmotor outcomes include neuropsychiatric symptoms (anxiety, depression, fatigue, and sleep disorders), autonomic dysfunction (sexual dysfunction, constipation, bladder issues, and fecal incontinence), sensory problems (pain), and reduced social participation.^3,4,5,6^ Over 50% of stroke survivors report these as significant long-term unmet needs.^3^ Despite guidelines suggesting integration of nonmotor outcomes into stroke care, data on their prevalence across multiple domains and natural progression over time are limited and inconsistent, with variation across studies.^6,7,8,9,10^ Effective clinical care requires a better understanding of these outcomes and their associated factors, which currently include factors such as age, sex, and stroke subtype, although findings are often contradictory.^11,12,13,14,15,16,17,18,19,20,21,22,23,24,25,26,27,28,29,30,31,32,33,34,35,36^ Comprehensive data on these nonmotor domains are needed to optimize patient-centered stroke care and address unmet needs.

To synthesize current published data on nonmotor outcomes after stroke and identify remaining knowledge gaps, we did a comprehensive systematic review and meta-analysis to determine the prevalence of various adverse nonmotor outcomes after stroke, including anxiety, depression, fatigue, sleep disturbance, social participation, pain, bowel dysfunction (constipation and fecal incontinence), bladder dysfunction, and sexual dysfunction. Additionally, we recorded the prevalence of each adverse nonmotor outcome at different time points to estimate their natural history over time. We also identified study-level factors associated with risk for each adverse nonmotor outcome.

## Methods

Using the Preferred Reporting Items for Systematic Reviews and Meta-analyses (PRISMA) reporting guidelines, we did a systematic review and meta-analysis with a preregistered PROSPERO protocol (CRD42019136337).^37^ We adhered to ethical standards, with all included studies following the Declaration of Helsinki. In this study, no individual patient data were collected, so no additional ethical approval was required.

### Literature Search and Study Selection

After consulting with a librarian, we searched PubMed, Embase, Medline via PubMed, PsycINFO, conference abstracts for English articles describing the prevalence of each nonmotor outcome (anxiety, depression, fatigue, sleep disturbance, social participation, pain, bladder dysfunction, bowel dysfunction [constipation and fecal incontinence], and sexual dysfunction) defined by patient-reported measures after acute ischemic stroke or intracerebral hemorrhage (ICH), time to follow-up, and associated clinical and sociodemographic factors from January 1, 1999, to June 30, 2023. For full search terms and mesh search words please see eTable 1 in Supplement 1.

### Selection Criteria

Two investigators (H.O. and T.E.) independently screened the literature eligibility, resolving discrepancies with senior authors (R.J.S. and D.J.W.) via consensus as required. When data were missing, we contacted study authors for raw data; 2 studies with unresolved queries more than 8 weeks after initial contact with the corresponding author were excluded.

We included peer-reviewed, English-language studies involving adults (aged ≥18 years) with ischemic stroke or ICH, reporting nonmotor outcomes at least 30 days after stroke. We included cohort, cross-sectional, and case-control studies, excluding those with transient ischemic attacks, subarachnoid hemorrhage, traumatic brain injury, pediatric stroke, and case series under 10 patients; see eTable 2 in Supplement 1 for exclusion criteria.

### Data Extraction

Using a predesigned Excel version 16.89.1 (Microsoft) spreadsheet, we extracted the following information from eligible studies: study characteristics (name, authors, year, sample size, design, follow-up time, stroke type, nonmotor outcome domain, outcome measure, and follow-up method), participants’ characteristics (sex and age), main outcomes (nonmotor outcomes 30 days after stroke), and analysis plan (statistical models and covariates).

### Statistical Analysis

We calculated the mean prevalence for each adverse nonmotor outcome at all time points for which it was reported. We assessed interrater reliability for study inclusion using the Cohen κ statistic. We assessed the quality of the included studies using the Newcastle-Ottawa Scale, a tool for evaluating nonrandomized studies.^290^ We performed a meta-analysis of all nonmotor domains for prevalence estimates, reported as proportion (%) with 95% CI. The heterogeneity between studies included in each nonmotor domain was assessed using the *I*^2^ and *H* statistics, with an *I*^2^ of more than 75% interpreted as indicating substantial heterogeneity. The heterogeneity ranged between 52% to 98% across all studies. To investigate publication bias, we performed funnel plots and meta-regression, considering study-level factors (see eFigures 1 and 2 in Supplement 1).

We performed meta-regression analysis to identify associations between study-level characteristics and the prevalence of adverse of nonmotor outcomes. The covariates in the meta-regression included: stroke type (ischemic stroke or ICH), sex, age group, and study design. We reported odds ratios (ORs) and 95% CIs for factors linked to adverse outcomes, and statistical significance was determined at a threshold of *P*=.05.

To explore the natural history of nonmotor outcomes, we performed a further meta-regression analysis to model the effect of time to follow-up after stroke on prevalence for each nonmotor outcome. The statistical significance between time to follow-up and adverse non-motor outcome prevalence was determined at threshold of *P*=.05. All statistical analyses were performed using Stata version 18 (StataCorp).

## Results

### Literature Search

The search strategy identified 11 601 unique citations. Initially, 817 studies met the inclusion criteria based on titles and abstracts. After further screening, 532 were excluded per criteria shown in eFigure 3 in in Supplement 1, leaving 279 studies^6,18,21,24,28,29,32,38,39,40,41,42,43,44,45,46,47,48,49,50,51,52,53,54,55,56,57,58,59,60,61,62,63,64,65,66,67,68,69,70,71,72,73,74,75,76,77,78,79,80,81,82,83,84,85,86,87,88,89,90,91,92,93,94,95,96,97,98,99,100,101,102,103,104,105,106,107,108,109,110,111,112,113,114,115,116,117,118,119,120,121,122,123,124,125,126,127,128,129,130,131,132,133,134,135,136,137,138,139,140,141,142,143,144,145,146,147,148,149,150,151,152,153,154,155,156,157,158,159,160,161,162,163,164,165,166,167,168,169,170,171,172,173,174,175,176,177,178,179,180,181,182,183,184,185,186,187,188,189,190,191,192,193,194,195,196,197,198,199,200,201,202,203,204,205,206,207,208,209,210,211,212,213,214,215,216,217,218,219,220,221,222,223,224,225,226,227,228,229,230,231,232,233,234,235,236,237,238,239,240,241,242,243,244,245,246,247,248,249,250,251,252,253,254,255,256,257,258,259,260,261,262,263,264,265,266,267,268,269,270,271,272,273,274,275,276,277,278,279,280,281,282,283,284,285,286,287,288,289,291,292,293,294,295,296,297,298,299,300,301,302,303,304,305,306,307^ for meta-analysis. Agreement for study inclusion between the reviewers was strong (κ = 0.87; 95% CI, 0.74-0.96).

Of the 279 studies, 160 (57.3%) included only patients with ischemic stroke, 113 (40.5%) included mixed cohorts, and only 6 (2.2%) focused on ICH. Research on nonmotor outcomes in ICH, the most debilitating stroke type, remains limited: only 1 study addressed anxiety, 2 investigated depression, and 3 explored pain. In 10 of 113 mixed cohort studies (8.8%), stroke type was undefined. Only 20 studies^17,18,19,20,21,22,23,24,25,26,27,28,29,30,31,32,33,34,35,36^ (7.2%) examined multiple nonmotor outcomes, with data for each domain analyzed separately. Comprehensive individual level study summary characteristics and our quality assessment of included studies can be found in eTable 3 in Supplement 1. Regarding risk of bias, 197 studies (70.6%) had low risk, 54 (19.4%) moderate, and 28 (10.0%) high (see eTable 4 in Supplement 1).

### Study Characteristics

Characteristics of the 279 included studies^6,18,21,24,28,29,32,38,39,40,41,42,43,44,45,46,47,48,49,50,51,52,53,54,55,56,57,58,59,60,61,62,63,64,65,66,67,68,69,70,71,72,73,74,75,76,77,78,79,80,81,82,83,84,85,86,87,88,89,90,91,92,93,94,95,96,97,98,99,100,101,102,103,104,105,106,107,108,109,110,111,112,113,114,115,116,117,118,119,120,121,122,123,124,125,126,127,128,129,130,131,132,133,134,135,136,137,138,139,140,141,142,143,144,145,146,147,148,149,150,151,152,153,154,155,156,157,158,159,160,161,162,163,164,165,166,167,168,169,170,171,172,173,174,175,176,177,178,179,180,181,182,183,184,185,186,187,188,189,190,191,192,193,194,195,196,197,198,199,200,201,202,203,204,205,206,207,208,209,210,211,212,213,214,215,216,217,218,219,220,221,222,223,224,225,226,227,228,229,230,231,232,233,234,235,236,237,238,239,240,241,242,243,244,245,246,247,248,249,250,251,252,253,254,255,256,257,258,259,260,261,262,263,264,265,266,267,268,269,270,271,272,273,274,275,276,277,278,279,280,281,282,283,284,285,286,287,288,289,307,308^ are shown in eTable 5 in Supplement 1. The total number of participants with stroke included in the meta-analysis was 117 440 (median [IQR] age, 65 [50-79] years; 209/279 studies^6,18,21,24,28,29,32,38,39,40,41,42,43,44,45,46,47,48,49,50,51,52,53,54,55,56,57,58,59,60,61,62,63,64,65,66,67,68,69,70,71,72,73,74,75,76,77,78,79,80,81,82,83,84,85,86,87,88,89,90,91,92,93,94,95,96,97,98,99,100,101,102,103,104,105,106,107,108,109,110,111,112,113,114,115,116,117,118,119,120,121,122,123,124,125,126,127,128,129,130,131,132,133,134,135,136,137,138,139,140,141,142,143,144,145,146,147,148,149,150,151,152,153,154,155,156,157,158,159,160,161,162,163,164,165,166,167,168,169,170,171,172,173,174,175,176,177,178,179,180,181,182,183,184,185,186,187,188,189,190,191,192,193,194,195,196,197,198,199,200,201,202,203,204,205,206,207,208,209,210,211,212,213,214,215,216,217,218,219,220,221,222,223,224,225,226,227,228,229,230,231,232,233,234,235,236,237,238,239,240,241,242,243,244,245,246,247,248,249,250,251,252,253,254,255,256,257,258,259,260,261,262,263,264,265,266,267,268,269,270,271,272,273,274,275,276,277,278,279,280,281,282,283,284,285,286,287,288,289,307,308^ with a dominance of male participants). Of the included studies, 32^6,18,24,28,32,38,39,40,41,42,43,44,45,46,47,48,49,50,51,52,53,54,55,56,57,58,59,60,61,62^ (11.4%) investigated anxiety, 52^21,29,63,64,65,66,67,68,69,70,71,72,73,74,75,76,77,78,79,80,81,82,83,84,85,86,87,88,89,90,91,92,93,94,95,96,97,98,99,100,101,102,103,104,105,106,107,108,109,110,111^ (18.6%) depression, 48^21,28,66,112,113,114,115,116,117,118,119,120,121,122,123,124,125,126,127,128,129,130,131,132,133,134,135,136,137,138,139,140,141,142,143,144,145,146,147,148,149,150,151,152^ (17.2%) fatigue, 50^153,154,155,156,157,158,159,160,161,162,163,164,165,166,167,168,169,170,171,172,173,174,175,176,177,178,179,180,181,182,183,184,185,186,187,188,189,190,191,192,193,194,195,196,197,198^ (17.9%) sleep disturbance, 20^199,200,201,202,203,204,205,206,207,208,209,210,211,212,213,214,215,216^ (7.2%) social participation, 32^217,218,219,220,221,222,223,224,225,226,227,228,229,230,231,232,233,234,235,236,237,238,239,240,241,242,243,244,245,246,307^ (11.4%) pain, 8^247,248,249,250,251,252,253,254,308^ (2.9%) constipation, 3^250,255,256^ (1.1%) fecal incontinence, 17^257,258,259,260,261,262,263,264,265,266,267,268,269,270,271,272,273^ (6.1%) bladder dysfunction, and 17^274,275,276,277,278,279,280,281,282,283,284,285,286,287,288,289^ (6.1%) sexual dysfunction after stroke. Nonmotor outcomes were mostly measured using patient-reported scales. However, each nonmotor outcome domain was assessed using 12 to 27 different scales, highlighting significant variability in measurement approaches.

In the majority of the studies,^6,18,21,24,28,29,32,38,39,40,41,42,43,44,45,46,47,48,49,50,51,52,53,54,55,56,57,58,59,60,61,62,63,64,65,66,67,68,69,70,71,72,73,74,75,76,77,78,79,80,81,82,83,84,85,86,87,88,89,90,91,92,93,94,95,96,97,98,99,100,101,102,103,104,105,106,107,108,109,110,111,112,113,114,115,116,117,118,119,120,121,122,123,124,125,126,127,128,129,130,131,132,133,134,135,136,137,138,139,140,141,142,143,144,145,146,147,148,149,150,151,152,153,154,155,156,157,158,159,160,161,162,163,164,165,166,167,168,169,170,171,172,173,174,175,176,177,178,179,180,181,182,183,184,185,186,187,188,189,190,191,192,193,194,195,196,197,198,199,200,201,202,203,204,205,206,207,208,209,210,211,212,213,214,215,216,217,218,219,220,221,222,223,224,225,226,227,228,229,230,231,232,233,234,235,236,237,238,239,240,241,242,243,244,245,246,247,248,249,250,251,252,253,254,255,256,257,258,259,260,261,262,263,264,265,266,267,268,269,270,271,272,273,274,275,276,277,278,279,280,281,282,283,284,285,286,287,288,289,307^ the follow-up time ranged between 30 days to 10 years after stroke. However, it’s important to note that in most studies, nonmotor outcomes were measured at a single time point; only 7^17,21,26,29,64,158,217^ of 279 (2.5%%) included follow-up at 2 or more time points. Data extraction for the adjusted analysis was available from the majority of the studies providing sociodemographic and clinical data such as age, sex, stroke subtype, study design, and time from stroke onset.

### Nonmotor Outcome Prevalence, Natural History, and Associated Factors

**Figure 1.**
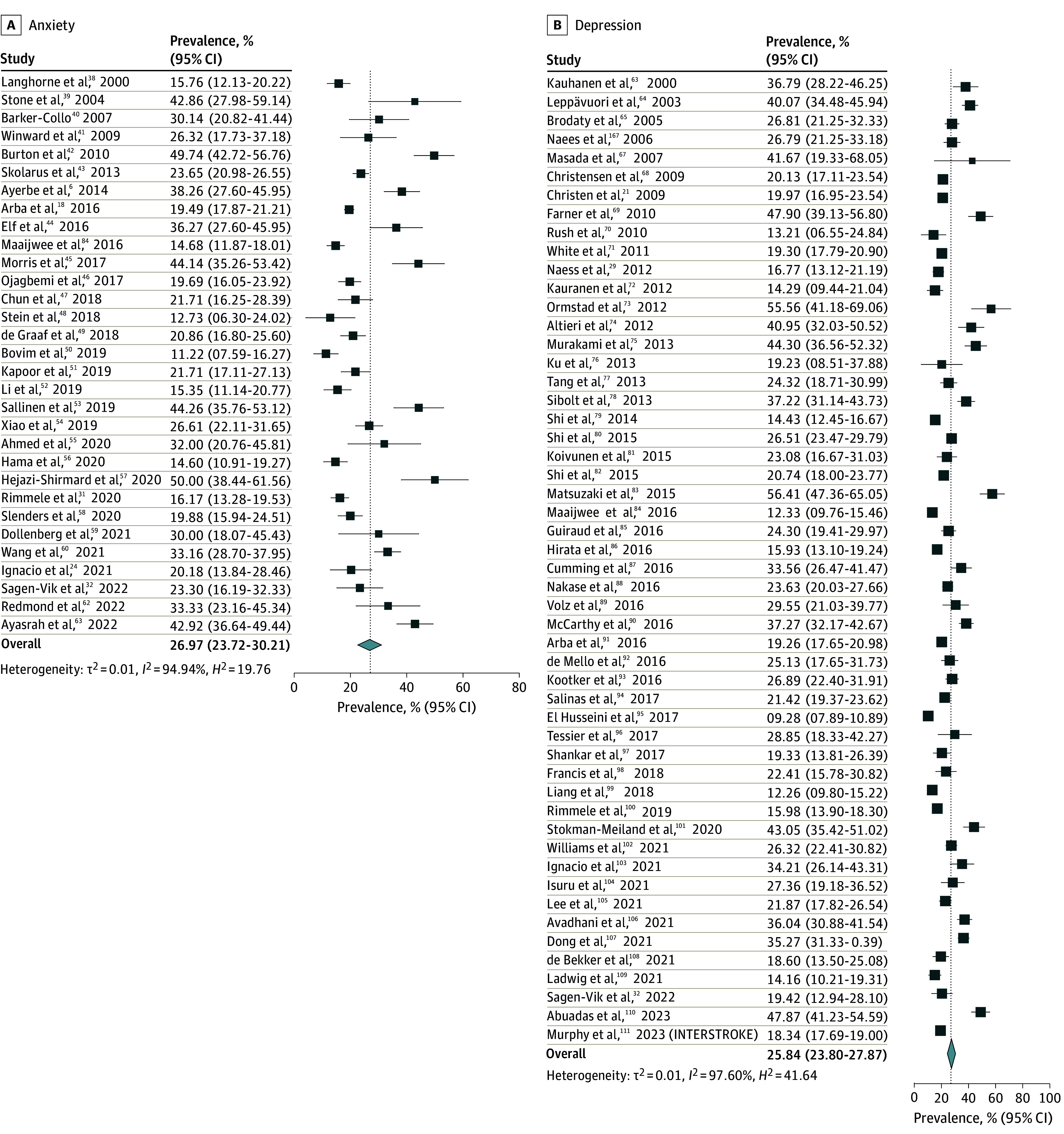
Pooled Prevalence of Anxiety and Depression The summary pooled prevalence estimates were calculated using a random effects model. Square markers represent each study’s prevalence estimate, with marker size reflecting the study’s weight (inverse variance of the effect estimate). The diamond marker illustrates the overall pooled estimate.

**Figure 2.**
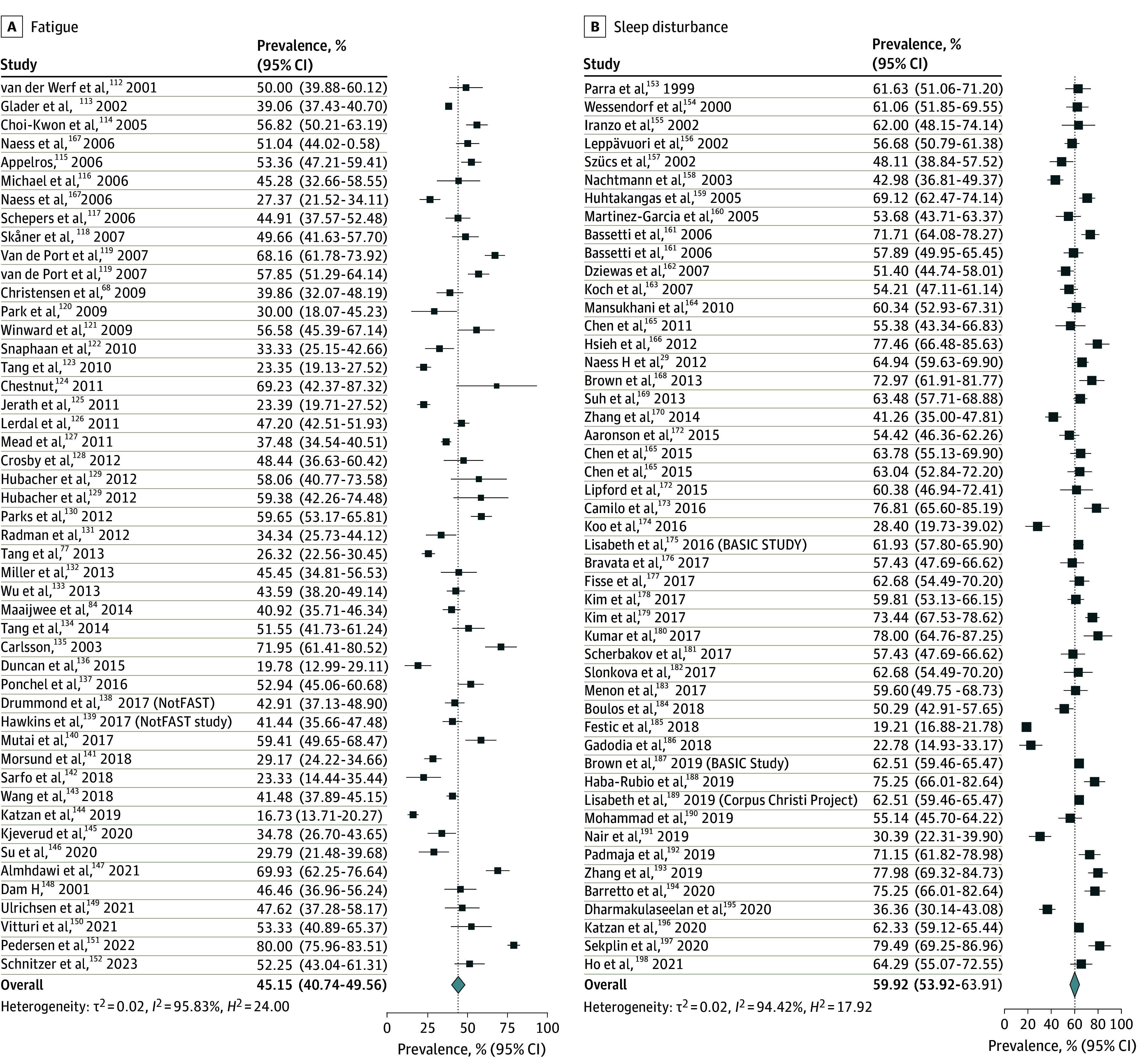
Pooled Prevalence of Fatigue and Sleep Disturbance The summary pooled prevalence estimates were calculated using a random effects model. Square markers represent each study’s prevalence estimate, with marker size reflecting the study’s weight (inverse variance of the effect estimate). The diamond marker illustrates the overall pooled estimate.

**Figure 3.**
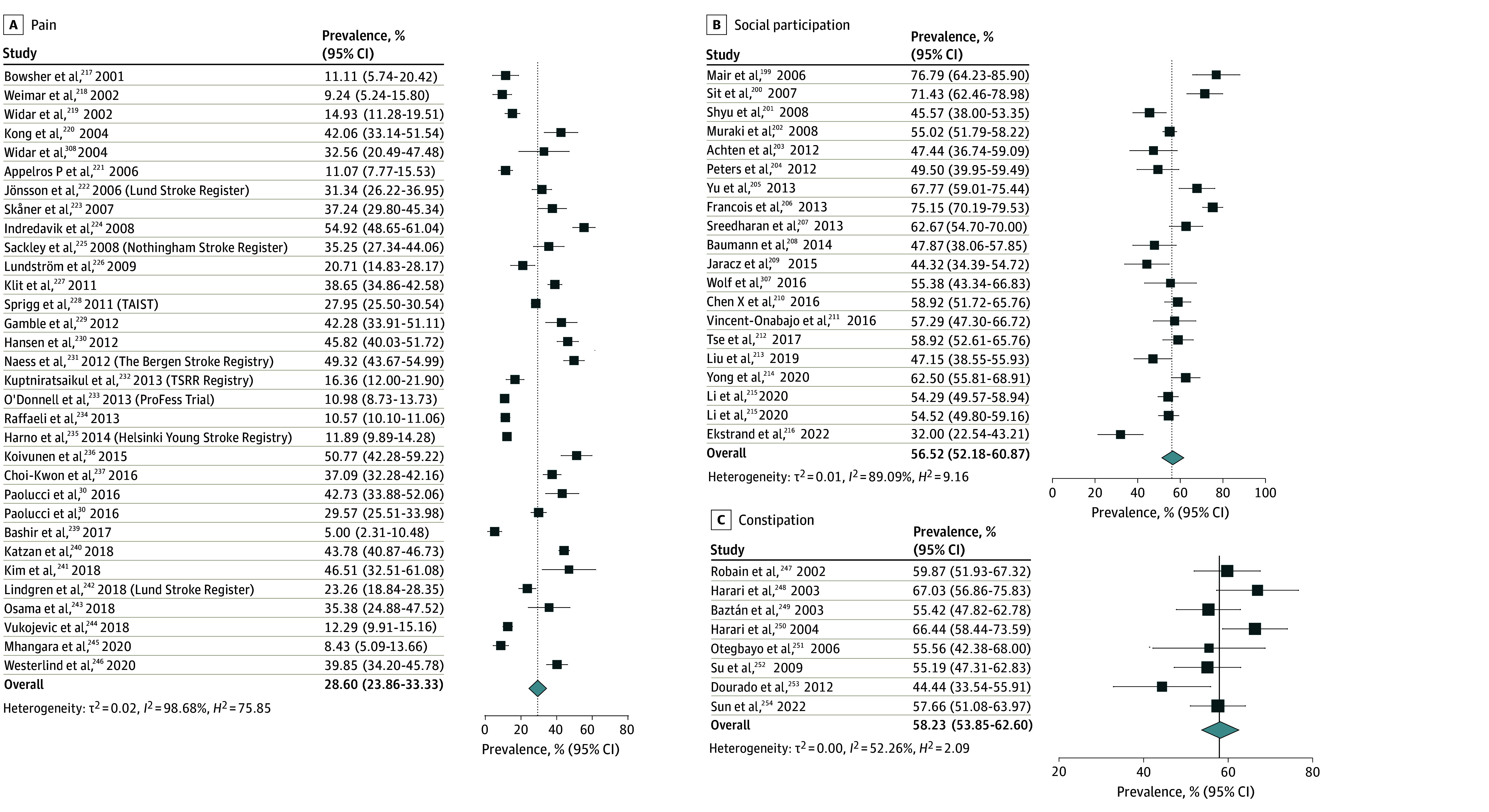
Pooled Prevalence of Pain, Social Participation, and Constipation The summary pooled prevalence estimates were calculated using a random effects model. Square markers represent each study’s prevalence estimate, with marker size reflecting the study’s weight (inverse variance of the effect estimate). The diamond marker illustrates the overall pooled estimate.

**Figure 4.**
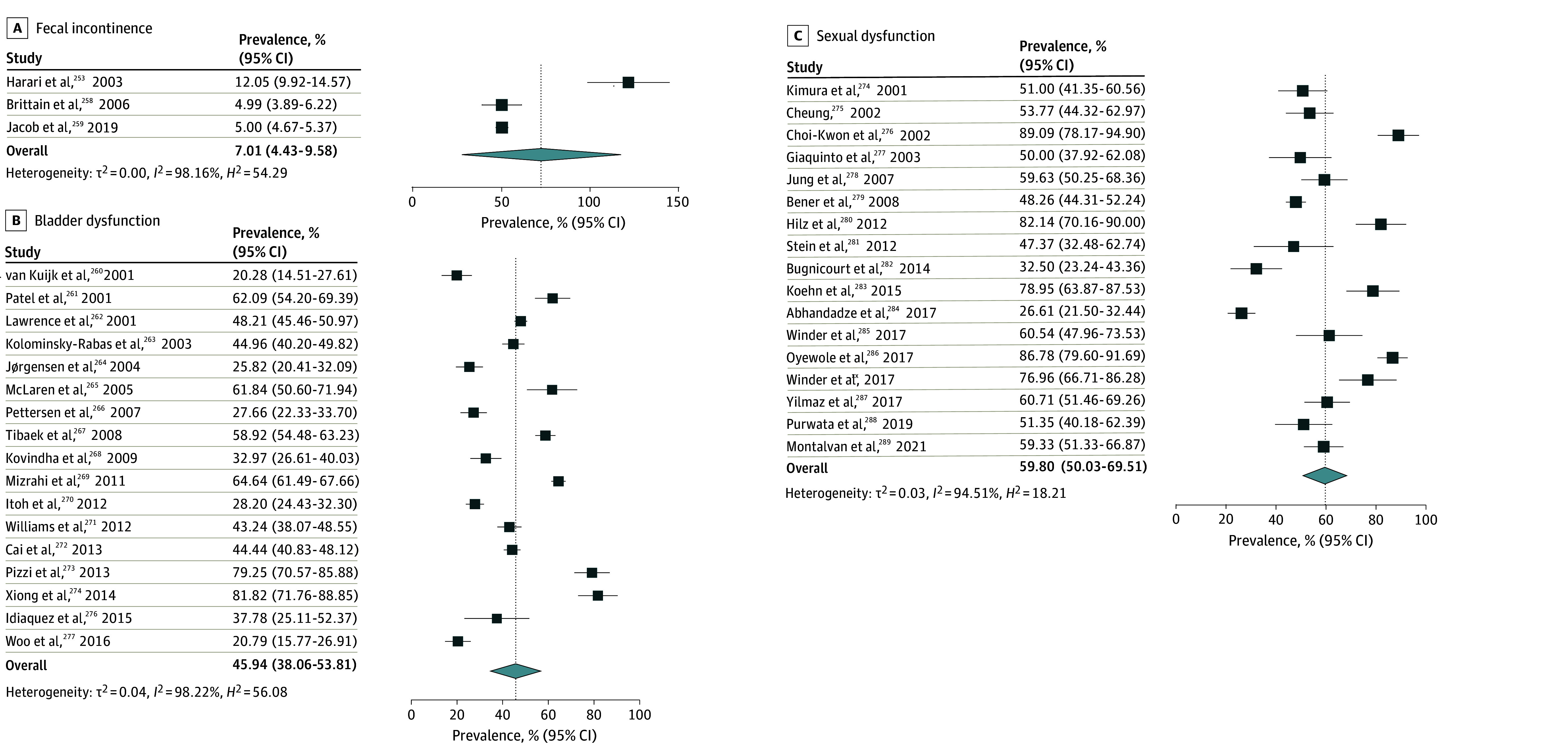
Pooled Prevalence of Fecal Incontinence, Bladder Dysfunction, and Sexual Dysfunction The summary pooled prevalence estimates were calculated using a random effects model. Square markers represent each study’s prevalence estimate, with marker size reflecting the study’s weight (inverse variance of the effect estimate). The diamond marker illustrates the overall pooled estimate.

The overall pooled prevalence of adverse nonmotor outcomes ranged between 59.9% (sleep disturbance) and 7.1% (fecal incontinence) across 10 domains (see Figures 1, 2, 3, and 4). Briefly, the most prevalent adverse nonmotor outcomes were sleep disturbance (59.9%; 95% CI, 53.9%-63.9%), sexual dysfunction (59.8%; 95% CI, 50.0%-69.5%), constipation (58.2%; 95% CI, 53.9%-62.6%), reduced social participation (56.5%; 95% CI, 52.1%-60.8%), bladder dysfunction (45.9%; 95% CI, 38.0%-53.8%), and fatigue (45.2%; 95% CI, 40.7%-49.5%). The least prevalent adverse nonmotor outcomes were fecal incontinence (7.0%; 95% CI, 4.4%-9.5%), depression (25.8%; 95% CI, 23.8%-27.8%), anxiety (26.9%; 95% CI, 23.7%-30.2%), and pain (28.6%; 95% CI, 23.6%-33.3%).

**Figure 5.**
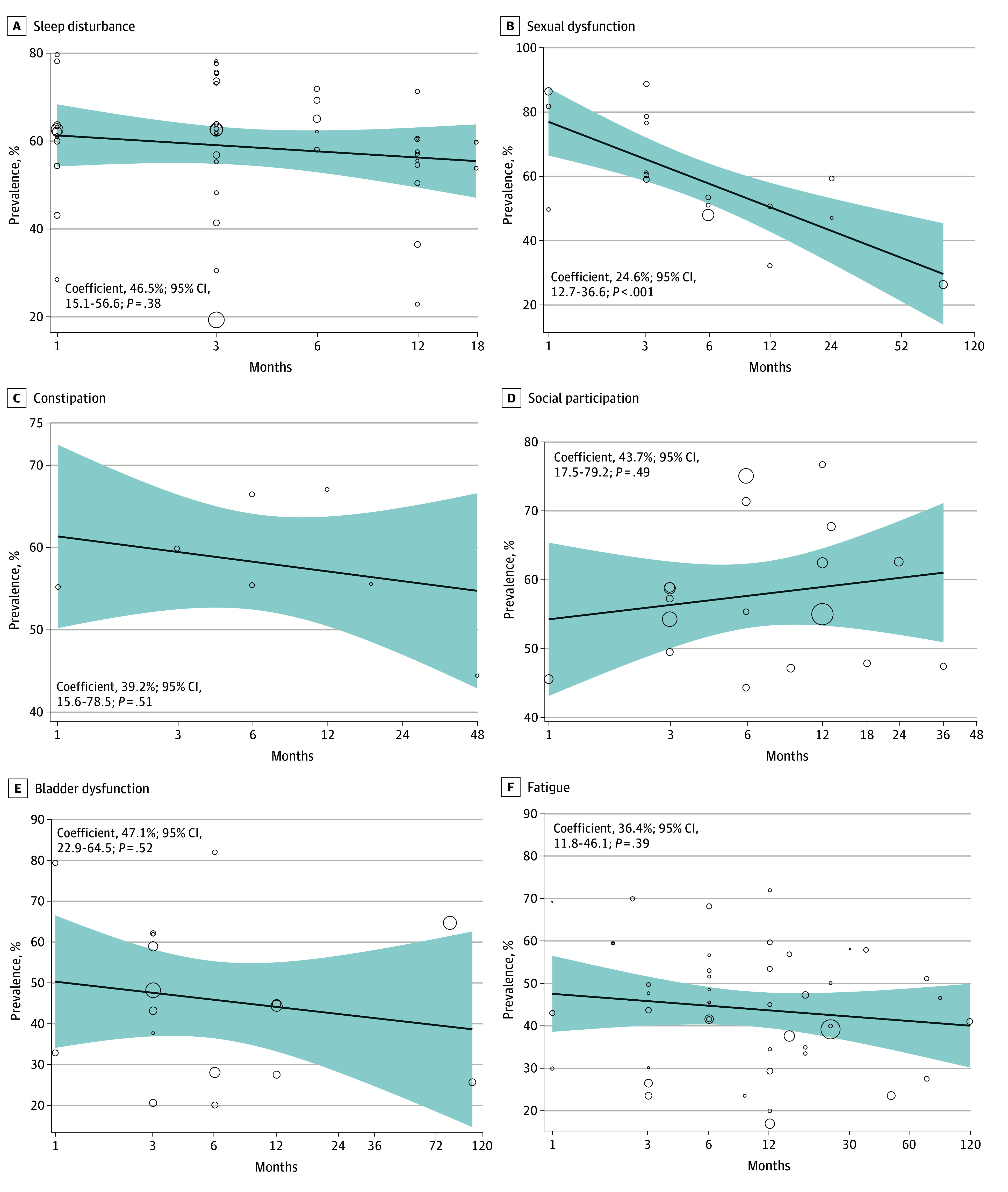
Natural History of Adverse Nonmotor Outcomes Time to follow-up adjusted meta-regression natural history graph adjusted for time to follow-up and the prevenance estimate reported in each study. The line represents the fitted regression model, while the shaded area indicates the 95% CI. Circles represent effect size of each study.

Figure 5 and eFigure 4 in Supplement 1 show meta-regression plots of prevalence according to the time to follow-up for all included studies conducted at different time points with defined study samples for each nonmotor domain. We found evidence of a statistically significant reduction in the prevalence of only 2 domains over time: pain (coefficient = 11.0%; 95% CI, 9.7%-22.1%; *P* = .05) (see eFigure 4 in Supplement 1) and sexual dysfunction (coefficient = 24.6%; 95% CI, 12.7%-36.6%; *P* < .001; see Figure 5B). We found no statistically significant reduction in adverse prevalence for the other 8 nonmotor outcome domains, including those that were most prevalent, such as sleep, constipation, social participation, and fatigue.

The results for the adjusted factors associated with of adverse nonmotor outcomes are shown in eTable 6 in Supplement 1. Briefly, the common factor associated with adverse nonmotor outcomes was mixed stroke cohort. We found that those studies with mixed cohorts including individuals with ischemic stroke and ICH were more likely to report higher odds of anxiety (OR, 2.06; 95% CI, 1.41-3.27; *P* = .01), fatigue (OR, 1.53; 95% CI, 1.16-1.75; *P* = .01), reduced social participation (OR, 2.69; 95% CI, 1.67-3.13; *P* = .01), pain (OR, 1.07; 95% CI, 1.01-2.63; *P* = .05), constipation (OR, 3.51; 95% CI, 1.70-4.21; *P* = .02), and sexual dysfunction (OR, 1.51; 95% CI, 1.28-2.88; *P* = .03) than the studies that only included ischemic stroke cohorts. Other study-level characteristics that were associated with at least 3 adverse nonmotor outcomes were older age, female sex, and hospital-based study design.

### Heterogeneity and Subgroup Analysis

The *I*^2^ statistic ranged from 52.26% to 98.22%, indicating substantial heterogeneity among studies within each nonmotor outcome domain. To investigate sources of heterogeneity, we conducted subgroup analyses on study-level characteristics (see eAppendix and eTable 7 in Supplement 1). Factors such as age, sex, stroke type, choice of outcome measures, and time to follow-up clarified heterogeneity in anxiety, depression, sleep disturbance, reduced social participation, constipation, fecal incontinence, and sexual dysfunction. For instance, studies on post-ICH anxiety, depression, and pain were generally less heterogeneous than those focusing on ischemic stroke or mixed cohorts.

Furthermore, we found that the heterogeneity in the domains of anxiety, sleep disturbance, pain, constipation, and sexual dysfunction could be explained by the type of assessment measures used. Additionally, variability in results for social participation, constipation, and sexual dysfunction could be attributed to the timing of follow-up assessments. However, we could not identify the sources of heterogeneity for other domains, such as fatigue and bladder dysfunction.

Finally, we performed subgroup analyses on 4 nonmotor domains with subdomain symptom heterogeneity: sleep disturbance, pain, bladder dysfunction, and sexual dysfunction. Our findings suggested that heterogeneity could be attributed to specific subdomain symptoms in sleep disturbance, with sleep-disordered breathing demonstrating less heterogeneity compared with obstructive sleep apnea and insomnia. In contrast, sources of heterogeneity for bladder dysfunction and other domains remained unexplained (see eAppendix, eTable 5, and eFigure 5 in Supplement 1).

## Discussion

We described a comprehensive systematic review and meta-analysis of poststroke adverse nonmotor outcomes, including 279 studies^6,18,21,24,28,29,32,38,39,40,41,42,43,44,45,46,47,48,49,50,51,52,53,54,55,56,57,58,59,60,61,62,63,64,65,66,67,68,69,70,71,72,73,74,75,76,77,78,79,80,81,82,83,84,85,86,87,88,89,90,91,92,93,94,95,96,97,98,99,100,101,102,103,104,105,106,107,108,109,110,111,112,113,114,115,116,117,118,119,120,121,122,123,124,125,126,127,128,129,130,131,132,133,134,135,136,137,138,139,140,141,142,143,144,145,146,147,148,149,150,151,152,153,154,155,156,157,158,159,160,161,162,163,164,165,166,167,168,169,170,171,172,173,174,175,176,177,178,179,180,181,182,183,184,185,186,187,188,189,190,191,192,193,194,195,196,197,198,199,200,201,202,203,204,205,206,207,208,209,210,211,212,213,214,215,216,217,218,219,220,221,222,223,224,225,226,227,228,229,230,231,232,233,234,235,236,237,238,239,240,241,242,243,244,245,246,247,248,249,250,251,252,253,254,255,256,257,258,259,260,261,262,263,264,265,266,267,268,269,270,271,272,273,274,275,276,277,278,279,280,281,282,283,284,285,286,287,288,289,307,308^ (117 440 participants) assessed in 10 nonmotor outcome domains at time points from 30 days up to 10 years. Adverse nonmotor outcomes including sleep disturbance, sexual dysfunction, reduced social participation, constipation, bladder dysfunction, and fatigue were reported by 50% of individuals included in the meta-analysis. Adverse outcomes in domains including anxiety, depression, and pain were also common, affecting more than 1 in 5 patients. We found that 8 of 10 domains persisted with no statistically significant trend for reducing prevalence over up to 10 years’ follow-up; we only noted evidence for a reduction over time for pain and sexual dysfunction. Factors including mixed cohorts (ischemic stroke and ICH), being older than 55 years, female sex, and hospital-based studies were significantly associated with a higher prevalence of adverse nonmotor outcomes across multiple domains.

We found that neuropsychiatric outcomes—such as anxiety, depression, fatigue, and sleep disturbance—were associated with 25.8% to 59.9% of individuals and persisted up to 10 years poststroke. Our pooled anxiety estimate of 26.9% aligns with previous studies but is a more precise estimate.^6,18,24,28,32,38,39,40,41,42,43,44,45,46,47,48,49,50,51,52,53,54,55,56,57,58,59,60,61,62^ One previous meta-analysis^291^ indicated a prevalence of 18.7% to 24.2% among 22 262 patients with stroke, while another^292^ with 44 studies (5760 patients) found an 18% to 25% prevalence. However, previous research included TIA and excluded severely ill patients with stroke, limiting generalizability. With regard to the trends in prevalence over time from stroke, a review^11^ of 97 studies involving 22 262 stroke survivors from 2009 to 2018 indicated increasing anxiety rates: 15.5% within 1 month, 21.4% at 1 to 5 months, and 31.8% at 6 to 12 months, consistent with our findings. Our observed depression prevalence is consistent with a recent meta-analysis (25%-30% within 5 years after stroke).^21,29,63,64,65,66,67,68,69,70,71,72,73,74,75,76,77,78,79,80,81,82,83,84,85,86,87,88,89,90,91,92,93,94,95,96,97,98,99,100,101,102,103,104,105,106,107,108,109,110,111,293^ However, there is limited research on the natural history of poststroke depression. Moreover, we found a 45.2% prevalence for poststroke fatigue, aligning with previous reports (42%-53%), but offering more recent and precise estimates.^21,28,66,112,113,114,115,116,117,118,119,120,121,122,123,124,125,126,127,128,129,130,131,132,133,134,135,136,137,138,139,140,141,142,143,144,145,146,147,148,149,150,151,152,294^ The natural history of fatigue remains debated; while some studies indicate decreasing prevalence, others suggest persistence, supporting our findings.^128,294,295,296^

Despite being the most common adverse nonmotor outcome (59.9%), previous comprehensive studies are limited for all forms of poststroke sleep disturbance.^153,154,155,156,157,158,159,160,161,162,163,164,165,166,167,168,169,170,171,172,173,174,175,176,177,178,179,180,181,182,183,184,185,186,187,188,189,190,191,192,193,194,195,196,197,198^ However, the results of the subgroup analysis align with prior estimates for sleep-disordered breathing (66.8% vs 63.4%) but differ somewhat for insomnia (57.6% vs 44.6%) and sleep apnea (58% vs 72%) (see eFigure 4 in Supplement 1).^298^ It is important to highlight that in comparison with previous studies, our study investigated sleep disturbance across all descriptive domains including insomnia, sleep apnea, and sleep disordered breathing. Despite the descriptive nature of poststroke sleep problems, historically, poststroke sleep disturbance received minimal clinical attention (6%), with just 2% of individuals undergoing formal testing after stroke.^299^

Previous data on risk predictors of neuropsychiatric outcomes are conflicting; some studies suggest significant association with older age at stroke, others with young age at stroke for adverse prevalence risk.^6,18,21,23,24,28,29,32,38,39,40,41,42,43,44,45,46,47,48,49,50,51,52,53,54,55,56,57,58,59,60,61,62,63,64,65,66,67,68,69,70,71,72,73,74,75,76,77,78,79,80,81,82,83,84,85,86,87,88,89,90,91,92,93,94,95,96,97,98,99,100,101,102,103,104,105,106,107,108,109,110,111,112,113,114,115,116,117,118,119,120,121,122,123,124,125,126,127,128,129,130,131,132,133,134,135,136,137,138,139,140,141,142,143,144,145,146,147,148,149,150,151,152,153,154,155,156,157,158,159,160,161,162,163,164,165,166,167,168,169,170,171,172,173,174,175,176,177,178,179,180,181,182,183,184,185,186,187,188,189,190,191,192,193,194,195,196,197,198^ Similarly, gender associations vary, with some studies suggesting significant association between female sex with anxiety, depression, fatigue, and sleep disturbance,^21,31,50,71,90,91,94,98,117,148^ while others show male associations or none.^28,31,59,83,89,93,99,116,119,223^ However, common risk factors reported for anxiety, depression, fatigue, and sleep disturbance include physical disability, stroke severity, and cognitive impairment.^6,18,21,23,24,27,28,29,32,38,39,40,41,42,43,44,45,46,47,48,49,50,51,52,53,54,55,56,57,58,59,60,61,62,63,64,65,66,67,68,69,70,71,72,73,74,75,76,77,78,79,80,81,82,83,84,85,86,87,88,89,90,91,92,93,94,95,96,97,98,99,100,101,102,103,104,105,106,107,108,109,110,111,112,113,114,115,116,117,118,119,120,121,122,123,124,125,126,127,128,129,130,131,132,133,134,135,136,137,138,139,140,141,142,143,144,145,146,147,148,149,150,151,152,153,154,155,156,157,158,159,160,161,162,163,164,165,166,167,168,169,170,171,172,173,174,175,176,177,178,179,180,181,182,183,184,185,186,187,188,189,190,191,192,193,194,195,196,197,198^ Moreover, in several studies, atrial fibrillation, hypertension, and diabetes were associated with sleep disturbance.^159,161,168,173,183,259^ In our study, we found that stroke case-mix (ischemic stroke or mixed ischemic stroke and ICH), female sex, and hospital-based study design were significantly associated with adverse neuropsychiatric outcomes. Previous studies suggest significant overlap between neuropsychiatric outcomes that might suggest shared underlying pathophysiological mechanisms, but further studies are needed to better clarify these.^6,18,21,23,24,27,28,29,32,38,39,40,41,42,43,44,45,46,47,48,49,50,51,52,53,54,55,56,57,58,59,60,61,62,63,64,65,66,67,68,69,70,71,72,73,74,75,76,77,78,79,80,81,82,83,84,85,86,87,88,89,90,91,92,93,94,95,96,97,98,99,100,101,102,103,104,105,106,107,108,109,110,111,112,113,114,115,116,117,118,119,120,121,122,123,124,125,126,127,128,129,130,131,132,133,134,135,136,137,138,139,140,141,142,143,144,145,146,147,148,149,150,151,152,153,154,155,156,157,158,159,160,161,162,163,164,165,166,167,168,169,170,171,172,173,174,175,176,177,178,179,180,181,182,183,184,185,186,187,188,189,190,191,192,193,194,195,196,197,198^

We found that autonomic adverse nonmotor outcomes—sexual dysfunction (59.8%), constipation (58.2%), fecal incontinence (7.0%), and bladder dysfunction (45.9%)—were very common after stroke.^247,248,249,250,251,252,253,254,255,256,257,258,259,260,261,262,263,264,265,266,267,268,269,270,271,272,273^ To our knowledge, ours is the first comprehensive meta-analysis on these domains, helping to address knowledge gaps left by small cohort studies and subdomain-focused reviews. Our analysis reveals a slightly lower pooled estimate (75%) for sexual dysfunction compared with previous reports, but the prior systematic review study was descriptive without formal meta-analysis, making its findings less statistically robust.^299^ Notably, our meta-regression analysis demonstrated a significant improvement in sexual dysfunction from 13 months to 10 years after stroke, which might be attributed to potential access to established sexual health clinics,^301^ although these are more widely available to younger individuals (<55 years) while older adults, including stroke survivors, can face stigmatization regarding sexual health.^302,303^ Our study identified cohort studies in stroke survivors aged 43 to 64 years, potentially excluding older stroke survivors who might not see the same improvement over time. Additionally, our adjusted analysis suggests that studies including mixed cohorts (ischemic stroke and ICH) report a higher rate of sexual dysfunction compared with those focused on ischemic stroke.^274,275,276,277,278,279,280,281,282,283,284,285,286,287^

Previous meta-analyses on poststroke autonomic dysfunction are limited. One study reported a constipation prevalence of 45% to 48%, slightly lower than our pooled estimate of 53.9% to 62.6%, potentially due to differences in study inclusion, follow-up duration, or outcome measures. Our findings are consistent with higher constipation rates in studies including ICH cohorts.^304^ Regarding fecal incontinence, our study suggests it was less prevalent than other nonmotor outcomes but persisted over time, with no prior meta-analyses found. Bladder dysfunction was associated with approximately one-third of stroke survivors, with no improvement observed longitudinally.^257,258,259,260,261,262,263,264,265,266,267,268,269,270,271,272,273^ A prior systematic review^255^ reported a lower prevalence (12%-19%), which could be due to narrower symptom definitions and shorter follow-up periods (up to 1 month vs up to 10 years in our study). We also identified a significant association between female sex and increased prevalence of bladder dysfunction after stroke. This association could be justified by the impact of reproductive health events such as pregnancy, childbirth, and menopause affecting the bladder, urethra, and supporting muscles in female survivors of stroke.^229,230,231,232,233,234,235,236,237,238,239,240,241,242,243,244^

With regard to sensory nonmotor outcomes, we found a 28.6% prevalence of poststroke pain at 3 months to 10 years after stroke.^217,218,219,220,221,222,223,224,225,226,227,228,229,230,231,232,233,234,235,236,237,238,239,240,241,242,243,244,245,246^ Earlier estimates vary widely (8%-55%) due to diverse pain types and methodological differences.^215,216,217,218,219,220,221,222,223,224,225,226,227^ We focused on patient-reported descriptive nonmotor pain domains, excluding trauma or motor-related pain. The pain types investigated in our study were bodily pain, central poststroke pain, unexplained pain, and shoulder pain. We found that the prevalence of pain decreased significantly with increasing time to follow-up, with an 11.0% drop over time. The significant decrease in the prevalence of pain may be attributed to the presence of well-established clinical pathways and pharmaceutical drugs for pain management.^305,306^ We identified older age (>55 years) and the inclusion of people with ICH as being significantly associated with higher pain prevalence. Several studies indicate that the incidence of poststroke pain is associated with age, sex or lesion location in the thalamus or brainstem, but we did not identify consistent associated factors, suggesting a need for further research.^20,217,218,219,220,221,222,223,224,225,226,227,228,229,230,231,232,233,234,235,236,237,238,239,240,241,242,243,244,245,246^

Reduced social participation was associated with 56.5% of patients with no significant improvement between 1 month to 3 years after stroke,^199,200,201,202,203,204,205,206,207,208,209,210,211,212,213,214,215,216,307^ but we did not identify previous systematic studies of prevalence for comparison. A recent narrative review suggested that impaired lower limb function, anxiety, pain, fatigue, older age, and lack of carer support were among significant factors associated with reduced social participation after stroke,^306^ but we were not able to adjust for these factors. Observational evidence suggests that increased social participation in midlife and late life is linked to a 30 to 50% reduced risk of developing dementia.^202^ Therefore, there is an urgent need for large cohort studies to establish the prevalence and predictors of reduced social participation after stroke.

### Strengths and Limitations

Our study has important strengths. We provided comprehensive data on poststroke nonmotor outcomes, detailing prevalence, natural history, and factors associated with risk across multiple domains and follow-up time points up to 10 years. This allowed us to investigate how adverse nonmotor outcomes might change over time. To our knowledge, we provided the first meta-analyses for key domains, including social participation, sexual dysfunction, and bowel dysfunction.

Our study also has several limitations. First, 12 studies^43,114,154,201,217,238,239,242,243,265,277,286^ in our analysis lacked stroke subtype definitions, although excluding them did not significantly alter our prevalence estimates. Second, only 6 studies^21,26,27,84,105^ focused on ICH cohorts. Third, diverse assessment measures, with 96 distinct patient-reported scales used across 10 nonmotor outcomes, contributed to variability in estimated prevalence. Another limitation of this study is the high heterogeneity observed across all nonmotor outcome domains, which may affect the reliability of our findings, particularly given our reliance on patient-reported measures for these symptoms. Sensitivity analysis showed that assessment measures were significantly associated with nonmotor outcome domains, accounting for 24% to 45% of the heterogeneity in constipation, and sexual dysfunction domains. Most studies conducted only a single follow-up time point, potentially affecting our pooled estimates of how the prevalence of adverse nonmotor outcomes change between 30 days to 10 years.

## Conclusions

In this systematic review and meta-analysis, poststroke nonmotor outcomes were highly prevalent. The most commonly affected domains were sleep disturbance, sexual dysfunction, constipation, reduced social participation, and fatigue. We found no evidence of significant improvement over time in 8 of the 10 domains up to 10 years after stroke. We determined that older age, female sex, studies including both ischemic stroke and ICH subtypes, and hospital-based settings were associated with a higher rate of adverse nonmotor outcomes. The persisting high prevalence of most domains suggest a major unmet health care need; strategies to detect, prevent, and treat poststroke nonmotor outcomes are urgently needed across the stroke care pathway from hospital to community care settings. However, given the limitations of the studies we have included, large prospective cohort studies using validated multidomain nonmotor outcome measures at multiple follow-up time points over a long period are also needed to better understand the natural history of all poststroke nonmotor outcomes. Improved data collection across multiple domains would help clinical services develop effective strategies to detect, prevent, and manage the wide and complex range of adverse nonmotor outcomes after stroke.

## Notes

Supplement 1. **eTable 1. **Characteristics of the Included Studies **eTable 2. **Keywords Used to Search Electronic Journal Databases **eTable 3. **Reasons for Exclusion **eFigure 1. **Funnel Plots to Assess Publication Bias (Anxiety, Depression, Fatigue, Sleep Disturbance, Social Participation, Pain) **eFigure 2. **Funnel Plots to Assess Publication Bias (Constipation, Faecal Incontinence, Bladder Dysfunction, and Sexual Dysfunction **eFigure 3.** Study Flowchart **eTable 4. **Summary Characteristics of Included Studies (in Chronological Order for Each Nonmotor Domain) **eFigure 4. **Natural History of Nonmotor Outcomes (G Pain), (H Anxiety), (I Depression), (J Fecal Incontinence) **eAppendix. **Summary of Sources of Heterogeneity **eTable 5**. Quality Assessment Assessed Using the Modified Newcastle-Ottawa Scale **eTable 6. **Adjusted Study-Level Characteristics Associated With the Prevalence of Adverse Nonmotor Outcomes **eFigure 5. **Sources for Heterogeneity Assessed Using Random Effects Meta-Analysis Model (Subgroup-Analysis of Each Nonmotor Outcome With Subsymptoms) **eTable 7. **Subgroup Analysis of Study Level Characteristics

Supplement 2.
Data Sharing Statement
