# Supplementary material for: Prevalence, Trajectory, and Factors Associated With Patient-Reported Nonmotor Outcomes After Stroke: A Systematic Review and Meta-Analysis
Source: JAMA Netw Open. 2025 Feb 21;8(2):e2457447. doi: 10.1001/jamanetworkopen.2024.57447 (PMC11846016; doi:10.1001/jamanetworkopen.2024.57447)
Supplement: Supplement 1. — eTable 1. Characteristics of the Included Studies eTable 2. Keywords Used to Search Electronic Journal Databases eTable 3. Reasons for Exclusion eFigure 1. Funnel Plots to Assess Publication Bias (Anxiety, Depression, Fatigue, Sleep Disturbance, Social Participation, Pain) eFigure 2. Funnel Plots to Assess Publication Bias (Constipation, Faecal Incontinence, Bladder Dysfunction, and Sexual Dysfunction eFigure 3. Study Flowchart eTable 4. Summary Characteristics of Included Studies (in Chronological Order for Each Nonmotor Domain) eFigure 4. Natural History of Nonmotor Outcomes (G Pain), (H Anxiety), (I Depression), (J Fecal Incontinence) eAppendix. Summary of Sources of Heterogeneity eTable 5. Quality Assessment Assessed Using the Modified Newcastle-Ottawa Scale eTable 6. Adjusted Study-Level Characteristics Associated With the Prevalence of Adverse Nonmotor Outcomes eFigure 5. Sources for Heterogeneity Assessed Using Random Effects Meta-Analysis Model (Subgroup-Analysis of Each Nonmotor Outcome With Subsymptoms) eTable 7. Subgroup Analysis of Study Level Characteristics [file jamanetwopen-e2457447-s001.pdf]

Supplemental Online Content

Ozkan H, Ambler G, Esmail T, Banerjee G, Simister RJ, Werring DJ. Prevalence, natural history, and predictors of patient-reported nonmotor outcomes after stroke. *JAMA Netw Open*. 2025;8(1):e2457447. doi:10.1001/jamanetworkopen.2024.57447

eTable 1. Keywords used to search electronic journal databases
eTable 2. Reasons for exclusion
eFigure 1. Funnel plots to assess publication bias (Anxiety, Depression, Fatigue, Sleep Disturbance, Social Participation, Pain)
eFigure 2. Funnel plots to assess publication bias (Constipation, Faecal Incontinence, Bladder Dysfunction, and Sexual Dysfunction
eFigure 3. Study Flowchart
eTable 3. Summary characteristics of included studies (in chronological order for each nonmotor domain)
eFigure 4 Natural history of nonmotor outcomes (G Pain), (H Anxiety), I Depression), (J Fecal Incontinence)
eAppendix. Summary of sources of heterogeneity
eTable 4. Quality assessment assessed using the modified Newcastle-Ottawa scale
eTable 5. Characteristics of the Included Studies
eTable 6. Adjusted study-level characteristics associated with the prevalence of adverse nonmotor outcomes
eFigure 5. Sources for heterogeneity assessed using random effects meta-analysis model (subgroup-analysis of each nonmotor outcome with subsymptoms)
eTable 7. Subgroup analysis of study level characteristics

This supplemental material has been provided by the authors to give readers additional information about their work.

© 2025 Ozkan H et al. *JAMA Network Open*.

eTable 1. Keywords used to search electronic journal databases

Database	Key Term	Search Terms
PubMed and Medline via PubMed	Ischemic Stroke	"Ischemic stroke"[TW] AND "Anxiety"[TW] AND "humans"[All fields]; "Ischemic stroke"[TW] AND "Depression"[TW] AND "humans"[All fields];"ischemic stroke"[TW] AND "fatigue"[TW] AND "humans"[All fields]; "ischemic stroke"[TW] AND "sleep"[TW] AND "humans"[All fields]; "stroke"[TW] AND "social participation"[TW] AND "humans"[All fields]; "ischemic stroke"[TW] AND "pain"[TW] AND "humans"[All fields]; "ischemic stroke"[TW] AND "bowel"[TW] AND "continence" AND "humans"[All fields]; "ischemic stroke"[TW] AND "bladder"[TW] AND "urinary" AND "humans"[All fields];"ischemic stroke"[All Fields] AND "sexual dysfunction"[All Fields] AND "humans"[All fields];"ischemic stroke"[TW] AND "autonomic dysfunction"[TW] AND "sexual dysfunction"[TW] OR "bowel" OR "bladder"[TW] OR "pain"[TW] OR "mood"[TW] OR "erectile dysfunction"[TW]) AND "humans"[MeSH Terms]
	Ischaemic Stroke	"Ischaemic stroke"[TW] AND "Anxiety"[TW] AND "humans"[All fields]; "Ischaemic stroke"[TW] AND "Depression"[TW] AND "humans"[All fields];"ischaemic stroke"[TW] AND "fatigue"[TW] AND "humans"[All fields]; "ischaemic stroke"[TW] AND "sleep"[TW] AND "humans"[All fields]; "stroke"[TW] AND "social participation"[TW] AND "humans"[All fields]; "ischaemic stroke"[TW] AND "pain"[TW] AND "humans"[All fields]; "ischaemic stroke"[TW] AND "bowel"[TW] AND "continence" AND "humans"[All fields]; "ischemic stroke"[TW] AND "bladder"[TW] AND "urinary" AND "humans"[All fields];"ischaemic stroke"[All Fields] AND "sexual dysfunction"[All Fields] AND "humans"[All fields];"ischaemic stroke"[TW] AND "autonomic dysfunction"[TW] AND "sexual dysfunction"[TW] OR "bowel" OR "bladder"[TW] OR "pain"[TW] OR "mood"[TW] OR "erectile dysfunction"[TW]) AND "humans"[MeSH Terms]
	Stroke	"stroke"[TW] AND "Anxiety"[TW] AND "humans"[All fields]; "stroke"[TW] AND "Depression"[TW] AND "humans"[All fields];"stroke"[TW] AND "fatigue"[TW] AND "humans"[All fields]; "stroke"[TW] AND "sleep"[TW] AND "humans"[All fields]; "stroke"[TW] AND "social participation"[TW] AND "humans"[All fields]; "stroke"[TW] AND "pain"[TW] AND "humans"[All fields]; "stroke"[TW] AND "bowel"[TW] AND "continence" AND "humans"[All fields]; "stroke"[TW] AND "bladder"[TW] AND "urinary" AND "humans"[All fields];"stroke"[All Fields] AND "sexual dysfunction"[All Fields] AND "humans"[All fields];"stroke"[TW] AND

© 2025 Ozkan H et al. *JAMA Network Open*.

		"autonomic dysfunction"[TW] AND "sexual dysfunction"[TW] OR "bowel" OR "bladder"[TW] OR “pain”[TW] OR “mood”[TW] OR “erectile dysfunction”[TW]) AND "humans"[MeSH Terms]
	Ischaemic Stroke Subtypes (Cardioembolic)	"Cardioembolic"[TW] AND "Anxiety"[TW] AND "humans"[All fields]; "stroke"[TW] AND "Depression"[TW] AND "humans"[All fields];" Cardioembolic "[TW] AND "fatigue"[TW] AND "humans"[All fields]; " Cardioembolic "[TW] AND "sleep"[TW] AND "humans"[All fields]; " Cardioembolic "[TW] AND "social participation"[TW] AND "humans"[All fields]; " Cardioembolic "[TW] AND "pain"[TW] AND "humans"[All fields]; "Cardioembolic"[TW] AND "bowel"[TW] AND “continence” AND "humans"[All fields]; "Cardioembolic"[TW] AND "bladder"[TW] AND "urinary” AND "humans"[All fields];"Cardioembolic"[All Fields] AND "sexual dysfunction"[All Fields] AND "humans"[All fields];"Cardioembolic"[TW] AND "autonomic dysfunction"[TW] AND "sexual dysfunction"[TW] OR "bowel" OR "bladder"[TW] OR “pain”[TW] OR “mood”[TW] OR “erectile dysfunction”[TW]) AND "humans"[MeSH Terms]
	Ischaemic Stroke Subtypes (Large-Artery Atherosclerosis)	"Large-Artery Atherosclerosis"[TW] AND "Anxiety"[TW] AND "humans"[All fields]; "Large-Artery Atherosclerosis"[TW] AND "Depression"[TW] AND "humans"[All fields];"Large-Artery Atherosclerosis"[TW] AND "fatigue"[TW] AND "humans"[All fields]; "Large-Artery Atherosclerosis"[TW] AND "sleep"[TW] AND "humans"[All fields]; "Large-Artery Atherosclerosis"[TW] AND "social participation"[TW] AND "humans"[All fields]; "Large-Artery Atherosclerosis"[TW] AND "pain"[TW] AND "humans"[All fields]; "Large-Artery Atherosclerosis"[TW] AND "bowel"[TW] AND “continence” AND "humans"[All fields]; "Large-Artery Atherosclerosis"[TW] AND "bladder"[TW] AND "urinary” AND "humans"[All fields];"Large-Artery Atherosclerosis"[All Fields] AND "sexual dysfunction"[All Fields] AND "humans"[All fields];" Large-Artery Atherosclerosis"[TW] AND "autonomic dysfunction"[TW] AND "sexual dysfunction"[TW] OR "bowel" OR "bladder"[TW] OR “pain”[TW] OR “mood”[TW] OR “erectile dysfunction”[TW]) AND "humans"[MeSH Terms]
	Intracerebral Haemorrhage	" Intracerebral haemorrhage "[TW] AND "Anxiety"[TW] AND "humans"[All fields]; " Intracerebral haemorrhage "[TW] AND "Depression"[TW] AND "humans"[All fields];"Intracerebral haemorrhage"[TW] AND "fatigue"[TW] AND "humans"[All fields]; " Intracerebral haemorrhage "[TW] AND "sleep"[TW] AND "humans"[All fields]; " Intracerebral haemorrhage "[TW] AND "social participation"[TW] AND "humans"[All fields]; "Intracerebral haemorrhage"[TW] AND "pain"[TW] AND "humans"[All fields]; "Intracerebral haemorrhage"[TW] AND "bowel"[TW] AND “continence” AND "humans"[All fields]; " Intracerebral haemorrhage"[TW] AND "bladder"[TW] AND "urinary” AND "humans"[All fields];"Intracerebral haemorrhage"[All Fields] AND "sexual dysfunction"[All Fields] AND "humans"[All fields];"Intracerebral haemorrhage "[TW] AND "autonomic

© 2025 Ozkan H et al. *JAMA Network Open*.

		dysfunction"[TW] AND "sexual dysfunction"[TW] OR "bowel" OR "bladder"[TW] OR “pain”[TW] OR “mood”[TW] OR “erectile dysfunction”[TW]) AND "humans"[MeSH Terms]
	Deep Intracerebral Haemorrhage	"Deep haemorrhage "[TW] AND "Anxiety"[TW] AND "humans"[All fields]; "Deep haemorrhage "[TW] AND "Depression"[TW] AND "humans"[All fields];"Deep haemorrhage"[TW] AND "fatigue"[TW] AND "humans"[All fields]; " Deep haemorrhage "[TW] AND "sleep"[TW] AND "humans"[All fields]; " Deep haemorrhage "[TW] AND "social participation"[TW] AND "humans"[All fields]; "Intracerebral haemorrhage"[TW] AND "pain"[TW] AND "humans"[All fields]; "Deep haemorrhage"[TW] AND "bowel"[TW] AND “continence” AND "humans"[All fields]; "Deep haemorrhage"[TW] AND "bladder"[TW] AND "urinary” AND "humans"[All fields];"Deep haemorrhage"[All Fields] AND "sexual dysfunction"[All Fields] AND "humans"[All fields];"Deep haemorrhage "[TW] AND "autonomic dysfunction"[TW] AND "sexual dysfunction"[TW] OR "bowel" OR "bladder"[TW] OR “pain”[TW] OR “mood”[TW] OR “erectile dysfunction”[TW]) AND "humans"[MeSH Terms]
	Lobar Intracerebral Haemorrhage	"Lobar haemorrhage "[TW] AND "Anxiety"[TW] AND "humans"[All fields]; "Lobar haemorrhage "[TW] AND "Depression"[TW] AND "humans"[All fields];"Lobar haemorrhage"[TW] AND "fatigue"[TW] AND "humans"[All fields]; " Lobar haemorrhage "[TW] AND "sleep"[TW] AND "humans"[All fields]; "Lobar haemorrhage "[TW] AND "social participation"[TW] AND "humans"[All fields]; "Lobar haemorrhage"[TW] AND "pain"[TW] AND "humans"[All fields]; "Lobar haemorrhage"[TW] AND "bowel"[TW] AND “continence” AND "humans"[All fields]; "Lobar haemorrhage"[TW] AND "bladder"[TW] AND "urinary” AND "humans"[All fields];"Lobar haemorrhage"[All Fields] AND "sexual dysfunction"[All Fields] AND "humans"[All fields];"Lobar haemorrhage "[TW] AND "autonomic dysfunction"[TW] AND "sexual dysfunction"[TW] OR "bowel" OR "bladder"[TW] OR “pain”[TW] OR “mood”[TW] OR “erectile dysfunction”[TW]) AND "humans"[MeSH Terms]
PsycINFO and EMBASE via Ovid	Stroke	ft(stroke) AND ft(anxiety); ft(stroke) AND ft(depression); ft(stroke) AND ft(fatigue); ft(stroke) AND ft(sleep); ft(stroke) AND ft(social participation); ft(stroke) AND ft(pain); ft(stroke) AND ft(bowel); ft(stroke) AND ft(bladder); ft(stroke) AND ft(sexual dysfunction
	Ischaemic Stroke	ft(ischemic stroke) AND ft(anxiety); ft(ischemic stroke) AND ft(depression); ft(ischemic stroke) AND ft(fatigue); ft(ischemic stroke) AND ft(sleep); ft(ischemic stroke) AND ft(social participation); ft(ischemic stroke) AND ft(pain); ft(ischemic stroke) AND ft(bowel); ft(ischemic stroke) AND ft(bladder); ft(ischemic stroke) AND ft(sexual dysfunction)

© 2025 Ozkan H et al. *JAMA Network Open*.

	Cardioembolic	ft(Cardioembolic stroke) AND ft(anxiety); ft(Cardioembolic stroke)AND ft(depression); ft(Cardioembolic stroke) AND ft(fatigue); ft(Cardioembolic stroke) AND ft(sleep); ft(Cardioembolic stroke) AND ft(social participation); ft(Cardioembolic stroke) AND ft(pain); ft(Cardioembolic stroke) AND ft(bowel); ft(Cardioembolic stroke) AND ft(bladder); ft(Cardioembolic stroke) AND ft(sexual dysfunction)
	Intracerebral Haemorrhage	ft(Intracerebral Haemorrhage) AND ft(anxiety); ft(Intracerebral Haemorrhage)AND ft(depression); ft(Intracerebral Haemorrhage) AND ft(fatigue); ft(Intracerebral Haemorrhage) AND ft(sleep); ft(Intracerebral Haemorrhage) AND ft(social participation);ft(Intracerebral Haemorrhage) AND ft(pain); ft(Intracerebral Haemorrhage) AND ft(bowel); ft(Intracerebral Haemorrhage) AND ft(bladder); ft(Intracerebral Haemorrhage) AND ft(sexual dysfunction)
	Deep Intracerebral Haemorrhage	ft(Deep Intracerebral Haemorrhage) AND ft(anxiety); ft(Deep Intracerebral Haemorrhage)AND ft(depression); ft(Deep Intracerebral Haemorrhage) AND ft(fatigue); ft(Deep Intracerebral Haemorrhage) AND ft(sleep); ft(Deep Intracerebral Haemorrhage) AND ft(social participation); ft(Deep Intracerebral Haemorrhage) AND ft(pain); ft(Deep Intracerebral Haemorrhage) AND ft(bowel); ft(Deep Intracerebral Haemorrhage) AND ft(bladder); ft(Deep Intracerebral Haemorrhage) AND ft(sexual dysfunction)
	Lobar Intracerebral Haemorrhage	ft(Lobar Intracerebral Haemorrhage) AND ft(anxiety); ft(Lobar Intracerebral Haemorrhage)AND ft(depression); ft(Lobar Intracerebral Haemorrhage) AND ft(fatigue); ft(Lobar Intracerebral Haemorrhage) AND ft(sleep); ft(Lobar Intracerebral Haemorrhage) AND ft(social participation); ft(Lobar Intracerebral Haemorrhage) AND ft(pain); ft(Lobar Intracerebral Haemorrhage) AND ft(bowel); ft(Lobar Intracerebral Haemorrhage) AND ft(bladder); ft(Lobar Intracerebral Haemorrhage) AND ft(sexual dysfunction)

© 2025 Ozkan H et al. *JAMA Network Open*.

eTable 2. Reasons for exclusion

1.	Paediatric Studies (age <18 years)
2.	Transient Ischaemic Attack (TIA)
3.	Subarachnoid Haemorrhage (SAH)
4.	Interventional Studies
5.	Time to Follow-up <30 Days
6.	Motor Outcomes via modified Rankin Scale (mRS)
7.	Caregiver Outcome
8.	Prevalence Not Reported
10.	Systematic Review
11.	Editorial
12.	Risk Prediction
13.	Evaluation of Scale
14.	Feasibility Trial
15.	Service Model
16.	Case Series with Few Patients (<10)

© 2025 Ozkan H et al. *JAMA Network Open*.

eFigure 1. Funnel plots to assess publication bias (Anxiety, Depression, Fatigue, Sleep Disturbance, Social Participation, Pain)

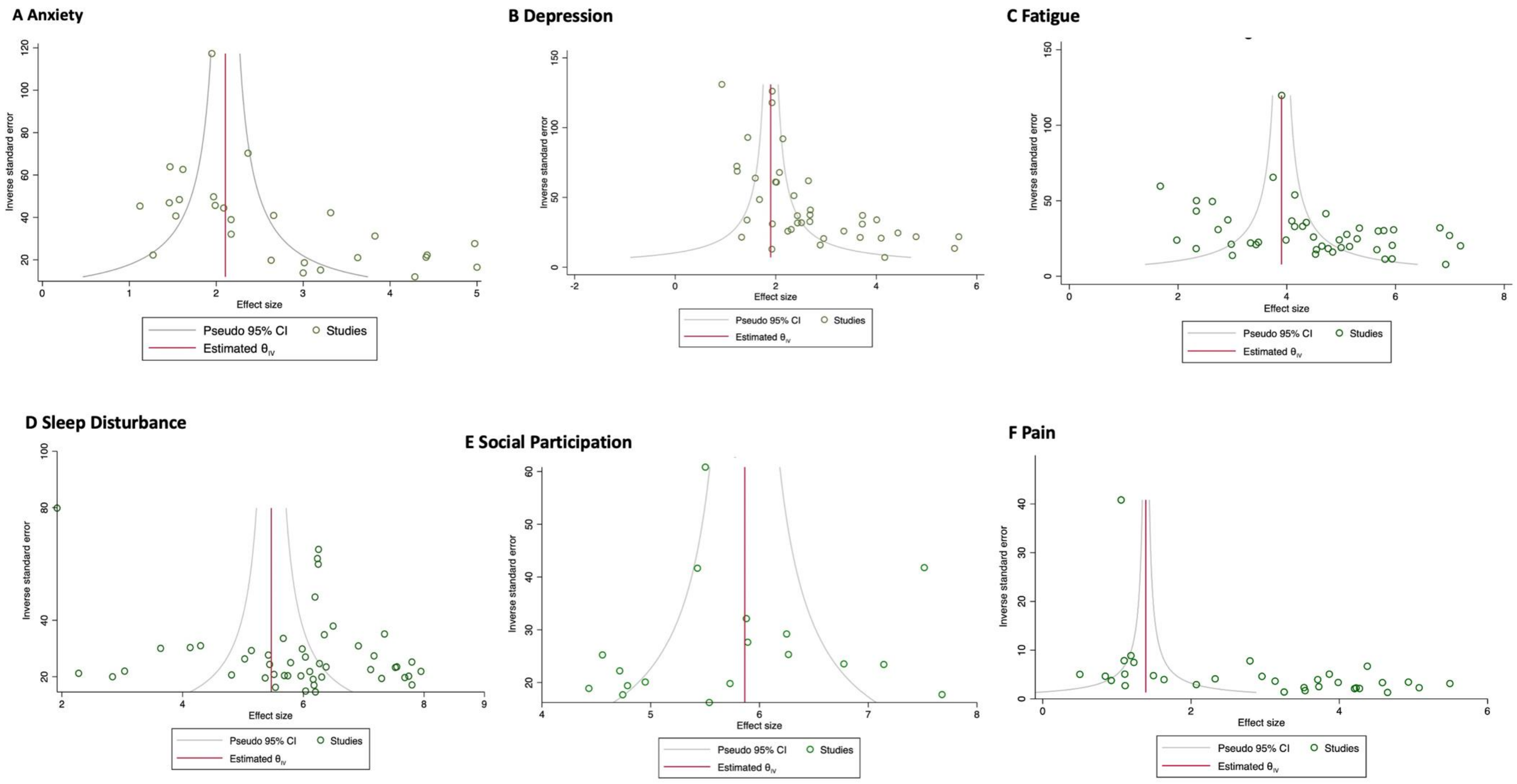

© 2025 Ozkan H et al. *JAMA Network Open*.

eFigure 2. Funnel plots to assess publication bias (Constipation, Faecal Incontinence, Bladder Dysfunction, and Sexual Dysfunction)

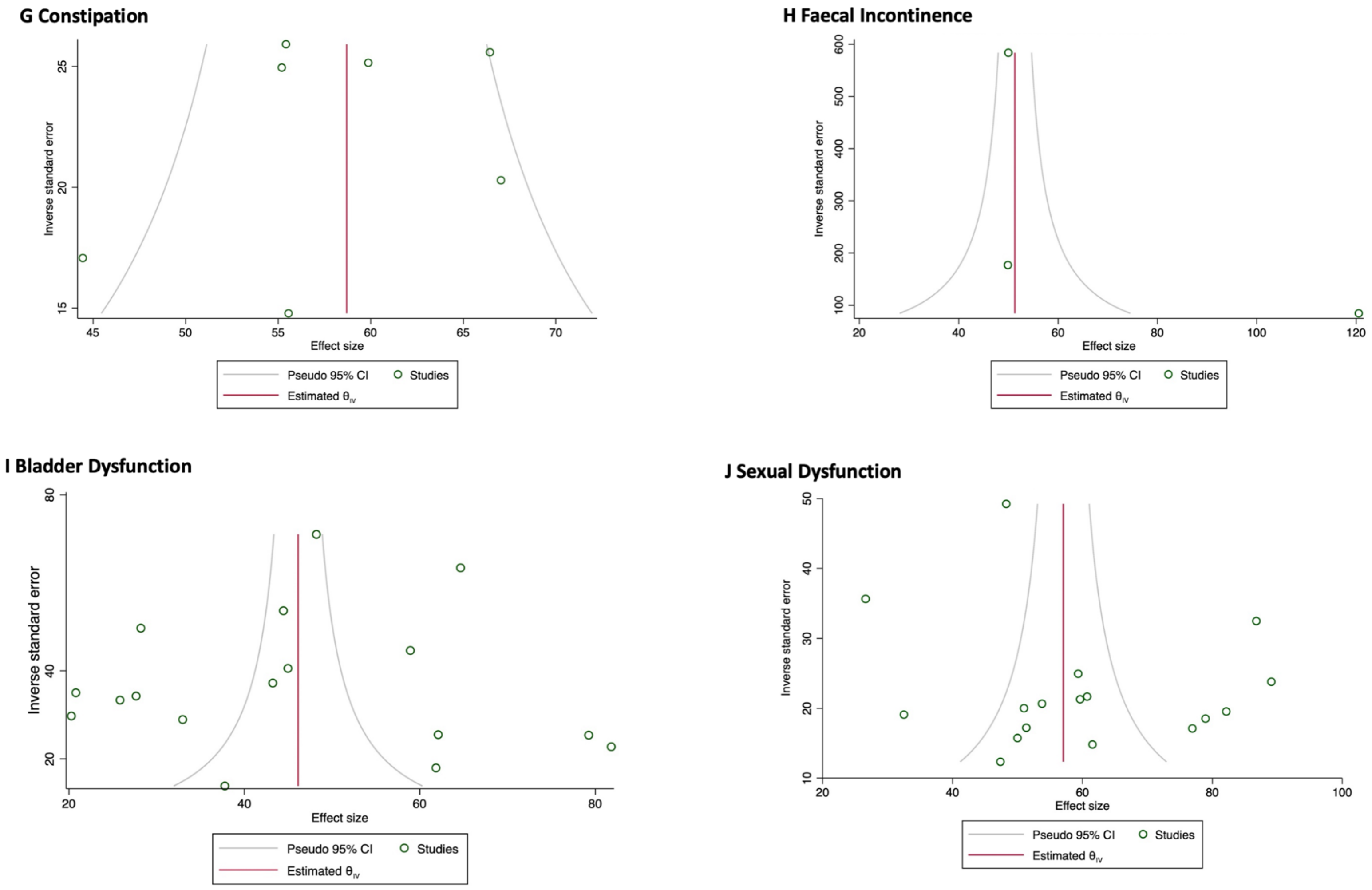

© 2025 Ozkan H et al. *JAMA Network Open*.

eFigure 3. Study Flowchart

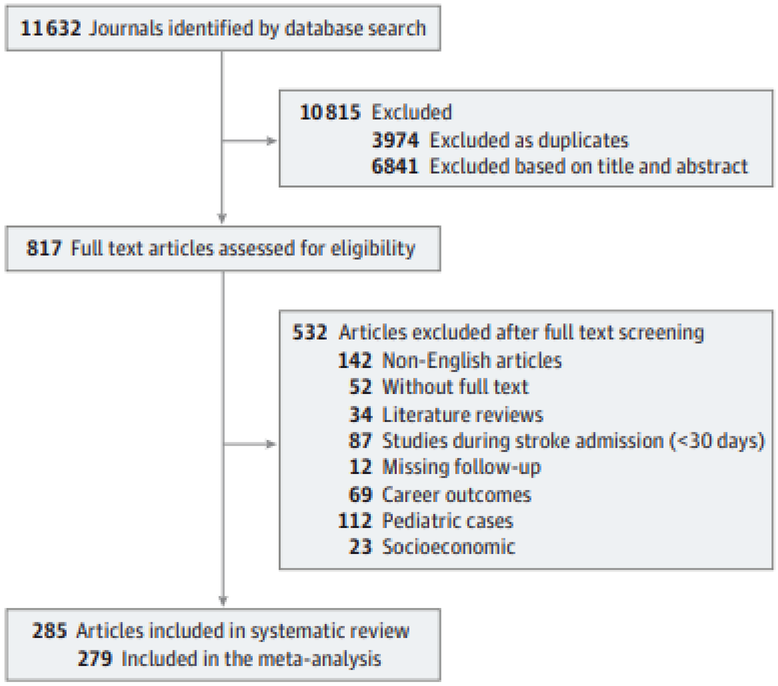

© 2025 Ozkan H et al. *JAMA Network Open*.

eTable 3. Summary characteristics of included studies (in chronological order for each nonmotor domain)

Author Year	Stroke Cohort	Number of patients	Number affected	Male (%)	Mean Age (In years*)	Study Design	Scale Used for Diagnosis	Prevalence (%)
**Anxiety**								
Langhorne et al.,2000	IS and ICH	265	37	52%	79	Hospital	Interview	14%
Stone et al.,2004	IS and ICH	35	15	69%	72	Hospital	HADS	42.9%
Barker-Collo., 2007	IS and ICH	72	22	54.8%	52	Hospital	BDI-II	30.6%
Winward et al., 2009	IS	76	20	58%	74	Population	N/A	26.3%
Burton et al., 2010	IS	191	95	52.4%	50	Hospital	SPARC	49.7%
Skolarus et al., 2013	IS	892	211	43%	72	Hospital	GAD	23.7%
Ayerbe et al., 2014	Stroke	230	88	38.3%	71	Population	HADS	38.3%
Arba et al., 2016	IS	2160	421	67%	64	Population	HADS	19.5%
Elf et al., 2016	IS and ICH	102	37	53%	62	Hospital	HADS	36.3%
Maaijwee et al., 2016	IS	511	75	25%	45	Hospital	HADS	23.0%
Morris et al., 2017	IS	111	49	69%	69	Hospital	HADS	44.1%
Ojagbemi et al., 2017	IS and ICH	391	77	63.7%	67	Population	HADS	19.7%
Stein et al., 2018	IS	55	7	55%	63	Hospital	HADS	12.7%
de Graaf et al., 2018	IS and ICH	326	68	65%	66	Hospital	HADS	20.9%
Chun et al., 2018	IS and ICH	175	38	60%	69	Population	Interview	21.7%
Bovim et al., 2019	IS and ICH	205	23	54%	74	Hospital	HADS	11.2%
Kapoor et al., 2019	IS and ICH	258	56	50%	65	Hospital	GAD7	21.7%
Sallinen et al.,2019	IS and ICH	122	54	64%	70	Hospital	EQ5D	44.3%

© 2025 Ozkan H et al. *JAMA Network Open*.

Xiao et al., 2019	IS	327	87	63%	60	Hospital	HAM-M	26.6%
Ahmed et al., 2020	IS	50	16	56%	56	Hospital	HADS	32%
Hama et al., 2020	IS	274	40	61%	64	Hospital	HADS	14.6%
Hejazi-Shirmard et al., 2020	IS and ICH	68	34	53%	55	Hospital	HADS	50%
Rimmele et al., 2020	IS and ICH	532	86	64.1%	71	Hospital	PROMIS	16.2%
Slenders et al., 2020	IS and ICH	332	66	64.5%	66	Hospital	HADS	19.9%
Wang et al., 2021	IS	395	131	69.9%	60	Hospital	HADS	33.2%
Dollenberg et al., 2021	Stroke	40	12	47.5%	70	Hospital	HADS	30%
Ayasrah et al., 2022	IS and ICH	226	97	57.1%	56	Hospital	Medical Notes	42.9%
Sagen-Vik et al., 2022	IS and ICH	103	24	53%	64	Hospital	GAD	23.3%
Redmond et al., 2022	IS and ICH	66	22	56%	66	Hospital	EQ-5D-3L	33.3%
Deb-Chartterji et al., 2022	Stroke	504	201	49%	76	Hospital	EQ-5D	39.9%
**Depression**								
Kauhanen et al., 2000	IS	106	39	75.5%	65	Hospital	Interview	36.8%
Leppavuori et al.,2003	IS	277	111	50.9%	70	Population	MADRs	40.1%
Naees et al., 2005	IS	209	56	50.7%	30	Hospital	MADRs	26.8%
Brodaty et al., 2005	IS	276	74	60.7%	72	Hospital	DSM-IV	26.8%
Masada et al., 2007	IS and ICH	12	5	48%	64	Hospital	N/A	41.7%
Christen et al., 2009	IS and ICH	596	119	60.3%	72	Population	HDRS	19.9%
Christensen et al., 2009	ICH	596	120	60.3%	70	Population	EuroQoL	20.1%
Farner et al., 2010	IS and ICH	119	57	54%	78	Hospital	MADRs	47.8%
Rush et al., 2010	IS	53	7	86.7%	75	Hospital	BDs	13.2%

© 2025 Ozkan H et al. *JAMA Network Open*.

White et al.,2011	IS	2477	478	63%	63	Hospital	PHQ-9	19.3%
Altieri et al., 2012	IS	105	43	64%	64	Hospital	BDI-SF	40.9%
Ormstad et al., 2012	IS	45	25	60%	67	Hospital	BDI	55.6%
Kauranen et al., 2012	IS	140	20	61%	65	Hospital	N/A	14.3%
Naess et al., 2012	IS	328	55	62.8%	67	Hospital	VAS	16.7%
Ku et al., 2013	IS	26	5	73.1%	60	Hospital	HADs	19.2%
Sibolt et al., 2013	IS	223	83	52%	71	Hospital	Interview	37.2%
Tang et al., 2013	IS	185	45	569%	69	Hospital	DSM-IV	24.3%
Murakami et al., 2013	IS and ICH	149	66	65%	66	Hospital	HADS	44.2%
Shi et al., 2014	IS	1067	154	68%	61	Population	Interview	14.4%
Koivunen et al., 2015	ICH	130	30	59.5%	42	Hospital	DSM-IV	23.1%
Matsuzaki et al., 2015	IS	117	66	45.3%	71	Hospital	SDS	56.4%
Shi et al., 2015	IS	747	198	68%	61	Population	Interview	26.5%
Shi et al., 2015	IS	757	157	67.7%	61	Population	Interview	26.5%
de Mello et al., 2016	IS and ICH	191	48	58.1%	56	Hospital	PHQ9	25.1%
Volz et al., 2016	IS	88	26	54.5%	66	Hospital	GSE	29.5%
Nakase et al., 2016	Stroke	474	112	65%	64	Hospital	QIDS-SR	23.6%
Maaijwee et al.,2016	IS	511	63	61%	40	Hospital	HADS	14.5%
Kootker et al.,2016	Stroke	331	89	36%	73	Hospital	DSM-IV	26.8%
Hirata et al., 2016	Stroke	546	87	48%	65	Hospital	PHQ9	15.9%
McCartney et al., 2016	IS	322	120	N/A	57	Hospital	MADRs	37.2%
Guiraud et al., 2016	IS	251	61	62%	71	Hospital	DSM-IV	19.4%

© 2025 Ozkan H et al. *JAMA Network Open*.

Cumming et al., 2016	IS and ICH	149	50	45%	81	Hospital	DSM-II	33.5%
Arba et al., 2016	IS	2160	416	67%	64	Population	HADS	19.3%
Husseini et al., 2017	IS	1444	134	61%	64	Hospital	HADS	9.2%
Salinas et al.,2017	IS	1424	305	45%	71	Hospital	HADS	21.4%
Shankar et al., 2017	IS and ICH	150	29	53%	66	Hospital	PHQ-2	19.3%
Tessier et al., 2017	IS	52	15	67%	51	Hospital	PHQ-8	28.8%
Liang et al., 2018	IS	563	69	59.2%	67	Hospital	GDS	12.2%
Francis et al., 2018	ICH	3422	140	48.4%	62	Population	PROMIS	22.4%
Liang et al., 2018	IS	563	69	49%	64	Hospital	HDRs	12.2%
Rimmele et al., 2020	IS and ICH	1064	170	52.2%	71	Hospital	PROMIS-10	15.9%
Stokman-Meiland et al., 2020	IS	151	65	58%	62	Hospital	Dutch Life Situation Cohort Questionnaire	43.04%
Williams and Deyemere., 2021	IS	437	115	51.7%	69	Hospital	HADS	26.3%
Ignacio et al., 2021	IS	114	39	57.9%	39	Hospital	HADS	34.2%
Isuru et al., 2021	IS	106	29	46%	60	Hospital	Interview	27.3%
Lee et al,. 2021	IS and ICH	343	75	53%	33	Hospital	Medical Notes	21.8%
Avadhani et al., 2021	ICH	308	111	61%	62	Hospital	CES-D	36.0%
Dong et al., 2021	IS	533	188	49.7%	68	Population	PHQ	32.5%
Bekker et al., 2021	IS	172	32	73%	63	Hospital	PHQ-9	18.6%
Ladwing et al., 2021	IS and ICH	226	32	54%	70	Hospital	Interview	14.1%
Sagen-Cik et al., 2022	IS	103	20	60%	64	Hospital	HADS	19.4%
Abuada et al., 2023	IS	211	101	52%	53	Hospital	Medical Records	47.9%
Murophy et al., 2023 (INTERSTOKE)	IS and ICH	2456	13392	59.6%	62	Population	DSM-4	18.3%

© 2025 Ozkan H et al. *JAMA Network Open*.

Fatigue								
van der Werf et al., 2001	IS and ICH	90	45	60%	62	Hospital	FSS	50%
Dam H., 2001	IS and ICH	99	46	63%	57	Hospital	Interview	46.5%
Glander et al., 2002	IS and ICH	3413	1333	62.9%	71	Hospital	Interview	39.1%
Naess et al., 2005	IS	192	98	57.3%	47	Hospital	FSS	51.4%
Choi-Kwon et al., 2005	IS and ICH	220	125	72%	59	Population	VAS	56.8%
Naess et al., 2006	IS	190	52	57%	29	Hospital	FSS	27.3%
Schepers et al., 2006	IS and ICH	167	75	48%	56	Hospital	FSS	44.9%
Appelros P., 2006	IS and ICH	253	135	50%	74	Population	FSS	53.3%
Michael et al., 2006	IS	53	24	54%	45	Population	FSS	45.3%
Skaner et al., 2007	IS and ICH	145	72	47%	73	Population	FSS	49.6%
Van de Port et al. 2007	IS	223	152	59.6%	57	Population	FSS	68.1%
Christensen et al., 2008	IS and ICH	138	55	56%	62	Population	MFI-20	39.8%
Park et al., 2009	IS and ICH	40	12	54%	59	Hospital	FSS	30%
Windward et al., 2009	IS	76	43	47%	74	Population	FSS	56.6%
Snaphaan L, Werf S.v.d, Leeuw., 2010	IS	108	36	69%	59	Hospital	Interview	33.3%
Tang et al., 2010	IS	334	78	54%	65	Hospital	FSS	23.3%
Mead et al., 2011	IS and ICH	1006	377	55.7%	71	Population	SF-36	37.4%
Lerdal A, and Kottorp A., 2011	IS	428	202	58%	68	Population	Interview	47.1%
Jerath et al., 2011	IS	449	105	56%	79	Hospital	Interview	23.3%
Chesnut T.J., 2011	IS	13	9	55%	68	Hospital	FSS	69.2%
Crosby et al., 2012	IS and ICH	64	31	53.1%	64	Hospital	FSS	48.4%

© 2025 Ozkan H et al. *JAMA Network Open*.

Hubacher et al.,2012	IS	32	19	58%	59	Population	FSS	59.3%
Parks et al., 2012	IS	228	136	47%	47	Hospital	FIS	59.6%
Radman et al., 2012	IS	99	34	67%	50	Population	Interview	34.3%
Tang et al., 2012	IS	475	125	61%	67	Hospital	FSS	26.3%
Wu et al., 2013	IS	312	136	57%	67	Hospital	FSS	43.5%
Miller et al., 2013	IS and ICH	77	35	54%	N/A	Hospital	Interview	45.4%
Tang et al., 2014	IS	97	50	57%	65	Hospital	FSS	51.5%
Maaijwee et al., 2014	IS	325	133	61%	50	Hospital	Interview	40.9%
Carlsson G.E., 2015	IS	82	59	53%	47	Hospital	Interview	71.9%
Duncan et al., 2015	IS	91	18	57%	71	Population	FAS-10	19.7%
Ponchel et al., 2016	IS	153	81	62%	64	Hospital	Interview	52.9%
Drummond et al., 2017 (NotFAST)	IS and ICH	268	115	52%	67	Hospital	FSS	42.9%
Hawkins et al., 2017	IS and ICH	263	108	52%	68	Hospital	FSS	41.4%
Mutai et al., 2017	IS	101	60	61%	74	Population	MFI	59.4%
Sarfo et al., 2018	IS and ICH	60	14	47%	54	Hospital	FSS	23.3%
Wang et al., 2018	IS	704	292	53%	59	Hospital	FSS	41.4%
Morsund et al., 2018	IS	288	84	57%	58	Population	FSS	29.1%
Katzan et al., 2019	IS and ICH	496	83	58%	61	Population	PROMIS	16.7%
Kjeverud et al., 2020	IS	115	40	62%	68	Population	FSS	34.7%
Su et al., 2020	IS and ICH	94	28	57%	70	Hospital	FSS	29.7%
Almhdawi et al., 2020	IS and ICH	153	107	53%	55	Hospital	MFI	69.9%
Dam et al., 2021	IS and ICH	60	32	54%	57	Hospital	FSS	46.5%

© 2025 Ozkan H et al. *JAMA Network Open*.

Vitturi et al., 2021	Stroke	99	46	51.7%	53	Hospital	FSS	53.3%
Ulrichsen et al., 2021	IS	84	40	56%	64	Hospital	FSS	47.6%
Almdawi et al., 2022	Stroke	153	107	69.6%	55	Hospital	Interview	69.9%
Pedersen et al.,2022	IS and ICH	430	344	65.5%	57	Hospital	D-FIS	80.0%
Shnitzer et al., 2023	Stroke	111	58	55.5%	70	Hospital	S-FAS	52.2%
**Sleep Disturbance**								
Parra et al., 1999	IS	86	53	100%	72	Hospital	Interview	62%
Wessendorf et al., 2000	IS	113	69	72.5%	58	Hospital	Polysomnography	61%
Szucs et al., 2002	ISH and ICH	106	51	64%	66	Hospital	MESAM IV	48%
Iranzo et L., 2002	IS	50	31	73%	66	Hospital	ESS	62%
Leppävuori et al., 2002	IS	277	157	52%	70	Hospital	DSM-IV	57%
Nachtman et al., 2003	IS	235	101	70%	66	Hospital	NETs	43%
Martinez et al., 2005	IS	95	51	64%	72.7	Hospital	ESS	54%
Huhtakangas et al., 2005	IS	204	141	67.3%	69	Hospital	ApneaLink	69%
Bassetti et al., 2006	IS	152	88	88%	59	Hospital	ESS	58%
Basetti C.L., Milanova M., and Gugger M., (2006)	IS	152	109	89%	64	Hospital	ESS	72%
Dziewas et al., 2007	IS	214	110	69%	67	Hospital	Polygraphy	51%
Koch et al., 2007	IS	190	103	53%	62	Hospital	BSQ	54%
Leino et al., 2010	IS	49	44	63%	69	Hospital	Polygraphy	90%

© 2025 Ozkan H et al. *JAMA Network Open*.

Mansukhani et al., 2010	IS	174	105	46%	71	Population	BSQ	60%
Chen et al., 2011	IS	65	36	44%	69	Population	Electroencephalography	55%
Hsieh et al., 2012	IS	71	55	66.4%	67	Hospital	ESS	77%
Naess H., Lunde L., Brogger J., 2012	IS	328	213	63%	67	Population	HRQoL	65%
Suh et al., 2013	IS	282	179	58.9%	62	Hospital	VSH	63%
Brown et al., 2013	IS	74	54	60%	N/A	Hospital	Polysomnography	73%
Tang et al., 2014	IS	113	13	53.8%	67	Hospital	REM sleep questionnaire	12%
Zhang et al., 2014	IS	223	92	76.2%	38	Hospital	PSQI	41%
Aaronson et al., 2015	IS	147	80	62.5%	60	Hospital	Polygraphy	54%
Chen et al., 2015	IS	92	58	72.4%	63	Hospital	ESS	63%
Lipford et al., 2015	IS	53	32	75%	67	Population	Polysomnography	60%
Chen et al., 2015	IS	127	81	76.7%	61	Hospital	Polysomnography	64%
Gupta et al., 2015	IS	349	43	68%	64	Hospital	Sleep Questionnaire	12%
Lisabeth et al., 2016 (BASIC STUDY)	IS	549	340	55%	65	Hospital	Cardiopulmonary monitoring device	62%
Koo et al., 2016	IS	81	23	66.6%	62	Population	Polysomnography	28%
Camilo et al.,2016	IS	69	53	75.4%	60	Hospital	Polysomnography	77%
Bravata et al., 2017	IS	102	58	99.1%	69	Hospital	Polygraphy	57%
Menon, Sukumaran, Varma, and Radhakrishnan., 2017	IS	99	59	68.7%	60	Hospital	Polysomnography	60%

© 2025 Ozkan H et al. *JAMA Network Open*.

Huhtakangas et al., 2017	IS	204	186	65.5%	65	Hospital	ESS	91%
Scherbakov et al., 2017	IS	101	58	61%	69	Population	Transthoracic impedance recording	57%
Kumar, Suri, and Manoca., 2017	IS &ICH	50	39	62%	54	Hospital	ESS	78%
Fisse et al., 2017	IS&ICH	142	89	64.7%	68	Hospital	Polygraphy	63%
Kim et al., 2017	IS	241	177	60.6%	60	Population	BSQ	73%
Kim et al., 2017	IS	214	128	48.4%	62	Population	KECA-R	60%
Slonkova et al., 2017	IS	68	42	47.1%	65	Hospital	Polygraphy	62%
Gadodia et al., 2018	IS	79	18	50%	60	Hospital	Interview	23%
Boulos et al., 2018	IS	173	87	71.1%	69	Hospital	Polysomnography	50%
Festic et al., 2018	IS	989	190	66%	73	Population	Polysomnography	19%
Zhang et al., 2019	IS	109	85	68.2%	63	Hospital	ESS	78%
Haba-Rubio et al., 2019	IS	101	76	84.1%	68	Hospital	Polysomnography	75%
Padmaja, Krishnaswamy, and Javali., 2019	IS & ICH	104	74	64.4%	60	Hospital	ESS	71%
Nair et al., 2019	IS	102	31	67.7%	71	Hospital	ESS	33%
Lisabeth et al., 2019 (Corpus Christi Project)	IS	995	622	61%	67	Population	ESS	63%
Brown et al., 2019 (BASIC Study)	IS	842	526	61.2%	65	Population	ApneaLink Plus	62%
Mohammad et al., 2019	IS	107	59	60%	63	Population	BSQ	55%
Dharmakulaseelan et al., 2020	IS	209	76	81.6%	70	Hospital	PEQ	36%

© 2025 Ozkan H et al. *JAMA Network Open*.

Barretto et al., 2020	IS	102	77	62.7%	59	Hospital	ESS	75%
Sekplin et al., 2020	IS	78	62	48.1%	59	Hospital	PSQI	79%
Katzan et al.,2020	IS and ICH	900	561	56.2%	60	Population	PROMIS-29	62%
Ho et al.,2021	IS	112	72	N/A	64	Hospital	VAS	64.3%
Schutz et al., 2022 (BASIC STUDY)	IS	1215	741	51%	68	Population	ApneaLink	64.4%
**Social Participation**								
Mair et al., 2006	IS	56	43	51.5%	57	Hospital	IADIq	76.7%
Sit et al., 2007	IS	112	80	52%	67	Population	SS	71.4%
Shyu et al., 2008	IS and ICH	158	72	61.5%	69	Population	ADL	45.6%
Muraki et al., 2008	IS	916	504	57%	70	Population	SS	55.1%
Achten et al., 2012	IS and ICH	78	37	37%	59	Hospital	LSS	47.4%
Petersen et al., 2012	IS	101	50	56.4%	47	Population	Interview	49.5%
Sreedharan et al., 2013	IS	150	94	65%	54	Population	SC	62.6%
Francois et al., 2013	IS	326	245	45%	73	Population	IC	75.1%
Yu et al., 2013	Stroke	121	82	62%	72	Hospital	MSPSS	67.7%
Baumann et al., 2014	IS	94	45	55%	65	Population	NSS	47.8%
Jaracz et al., 2015	IS	88	39	57%	61	Hospital	PSS	44.3%
Vincent-Onabajo et al., 2016	IS and ICH	96	55	62.5%	56	Population	SS	57.3%
Wolf et al., 2016	IS	65	36	51.6%	59	Hospital	SS	55.3%
Chen et al., 2016	IS	250	147	60.7%	68	Population	SS	58.8%
Tse et al., 2017	IS and ICH	185	109	87%	67	N/A	SS	58.9%
Liu et al., 2019	IS	123	58	53%	75	Population	SS	47.2%

© 2025 Ozkan H et al. *JAMA Network Open*.

Li et al., 2020	IS	431	234	65.7%	70	Hospital	PSS	54.2%
Li et al., 2021	Stroke	431	235	67.1%	70	Hospital	Interview	54.5%
Ekstrand and Brogardh., 2022	Stroke	75	24	72%	66	Hospital	Interview	32.0%
**Pain**								
Bowsher et al., 2001	IS and ICH	72	8	50%	77	Hospital	Interview	11%
Weimar et al., 2002	IS and ICH	119	11	64.7%	63	Hospital	SES	9%
Widar et al., 2002	IS and ICH	288	43	70%	66	Hospital	MSPI	15%
Kong et al., 2004	IS and ICH	107	45	64%	60	Hospital	BPI	42%
Widar et al., 2004	IS and ICH	43	14	70%	66	Hospital	SF-36	33%
Jonson et al., 2006)	IS and ICH	284	89	60%	71	Population	VAS	31%
Apperlos P,.2006	IS and ICH	253	28	N/A	76	Population	Interview	11%
Skaner et al., 2007	IS and ICH	145	54	48%	73	Hospital	GQLA	37%
Sackley et al., 2008	IS	122	43	57%	76	Population	Medical Notes	35%
Indredavik et al., 2008	IS and ICH	244	134	47.6%	78	Hospital	Interview	55%
Ludstrom et al., 2009	IS	140	29	52%	47	Hospital	VAS	21%
Klit et al., 2011	IS and ICH	608	235	56%	55	Population	CPSP	39%
Sprigg et al., 2011	IS	1220	341	N/A	70	Hospital	SF-36	28%
de Oliveiraet al., 2012	IS and ICH	39	31	65%	59	Hospital	VAS	79%
Naess et al., 2012	IS	296	146	60%	67	Population	VAS	49%
Hansen et al., 2012	IS and ICH	275	126	55.6%	65	Hospital	Interview	46%
Gamble et al., 2012	IS and ICH	123	52	48%	68	Population	VAS	42%

© 2025 Ozkan H et al. *JAMA Network Open*.

Kuptniratsaikul et al., 2013	IS and ICH	327	106	57.9%	62	Hospital	MAS	32%
O'Donnell et al., 2013	IS	15754	1665	65.7%	65	Hospital	Interview	11%
Raffaeli et al., 2013	IS and ICH	601	66	54.4%	76	Population	VAS	11%
Kuptniratsaikul et al., 2013	IS and ICH	214	35	57.9%	62	Hospital	Interview	16%
Harno et al., 2014	IS	824	98	N/A	41	Population	PainDETECT + EQ5D	12%
Koivunen et al., 2015	ICH	130	66	59.5%	42	Population	PIS	51%
Paolluci et al., 2016	IS and ICH	110	47	59.1%	67	Hospital	NPIS	43%
Choi-Kwon et al., 2016	IS	364	135	63.5%	60	Population	Interview	37%
Paolluci et al., 2016	IS	443	131	58.6%	66	Hospital	NPIS	30%
Bashir et al., 2017	IS	120	6	47.5%	55	Hospital	DN4	5%
Osama et al., 2018	IS	65	23	64.6%	53	Hospital	McGhill Pain Questionnaire	35%
Kim et al., 2018	ICH	43	20	N/A	58	Hospital	Sensory subscale of fugl-Meyer	47%
Vukojevic et al., 2018	IS and ICH	602	74	N/A	62	Hospital	Pain DETECT, LANSS scale	12%
Katzan et al., 2018	IS	1101	482	N/A	62	Population	PROMIS-29	44%
Katzan et al., 2018	ICH	215	192	N/A	59	N/A	PROMIS-29	89%
Lindgren et al., 2018	IS and ICH	301	70	60%	73	Hospital	VAS	23%
Westerlind et al., 2020	IS and ICH	271	108	60.9%	65	Population	EQ5D	40%
Mhangara et al., 2020	IS and ICH	166	14	30%	58	Hospital	LANSS	8%
**Constipation**								
Robain et al., 2002	IS	152	91	41%	62	Hospital	BI	59.8%

© 2025 Ozkan H et al. *JAMA Network Open*.

Harari et al., 2002	IS	91	61	48%	66	Hospital	BI	66.4%
Baztan et al., 2003	IS and ICH	166	92	53%	78	Hospital	BI	55.4%
Harari et al., 2004	IS and ICH	146	97	56%	72	Population	Interview	66.4%
Otegbayo et al., 2006	IS	54	30	42%	65	Hospital	Interview	55.5%
Su et al., 2009	IS and ICH	154	85	54%	41	Hospital	Rome Criteria II	55.1%
Dourado et al., 2012	IS and ICH	72	32	43%	46	Hospital	Interview	58.5%
Sun et al., 2022	IS	222	128	57%	64	Hospital	Barthel Index	57.6%
**Faecal Incontinence**								
Harari et al., 2002	IS and ICH	755	91	61%	70	Population	Survey	4.9%
Brittain et al., 2006	IS and ICH	1,483	74	57%	56	Hospital	Survey	4.9%
Jacop L., and Kostev K., 2019	IS and ICH	16,181	809	55%	67	Hospital	BI	12.1%
**Bladder Dysfunction**								
van Kujik et al., 2001	IS and ICH	143	29	74%	67	Hospital	BI ADL Index	20.2%
Patel et al., 2001	IS	153	95	N/A	73	Hospital	BI ADL Index	62.1%
Lawrance et al., 2001	IS and ICH	1259	607	46.8%	71	Registry	BI	48.2%
Kolominsky-Rabas et al., 2003	IS	407	183	54%	73	Registry	BI	44.9%
Jorgensen et al., 2004	IS and ICH	213	55	58%	67	Population	ISI	25.8%
McLaren et al., 2005	IS	76	47	42%	80	Hospital	Valsava Ratio	61.8%
Pettersen et al., 2007	IS	235	65	56%	62	Hospital	BI	27.6%
Tibaek et al., 2008	IS	482	284	N/A	71	Hospital	DAN-PSS-1	58.9%
Mizrah et al., 2011	IS and ICH	919	594	55.9%	76	Hospital	FIM Bladder	64.6%
Itoh et al., 2012	IS and ICH	500	141	70.9%	73	Hospital	QABSS	28.2%

© 2025 Ozkan H et al. *JAMA Network Open*.

Pizzi et al., 2013	IS	106	84	64%	72	Hospital	BI	79.2%
Cai et al., 2013	IS and ICH	711	316	67%	65	Survey	Interview	44.4%
Xiong et al., 2014	IS	77	63	48.6%	69	Hospital	Ewing's Battery	81.8%
Idiaquez et al., 2015	IS	45	17	62.2%	65	Hospital	AUT	37.7%
Woo et al., 2016	IS and ICH	202	42	55%	69	Hospital	BI	20.8%
**Sexual Dysfunction**								
Kimura et al., 2001	IS and ICH	100	51	58.6%	60	Hospital	Interview	51%
Choi-Kwon S, and Kim J.S., 2002	IS	55	49	N/A	56	Population	Interview	89.0%
Cheung R.t.F., 2002	IS and ICH	106	57	51.6%	56	Hospital	Interview	53.7%
Giaquinto et al., 2003	IS and ICH	62	31	N/A	64	Hospital	Interview	50%
Jung et al., 2007	IS and ICH	109	65	100%	64	Hospital	IIEF	59.6%
Bener et al., 2008	IS and ICH	605	292	100%	62	Hospital	IIEF	48.2%
Hilz et al., 2012	IS	56	46	100%	64	Hospital	IIEF	82.1%
Stein et al., 2012	IS and ICH	38	18	77.8%	55	Hospital	Interview	47.3%
Bugnicourt et al., 2014	IS	80	26	100%	48	Hospital	Interview	32.5%
Koehn et al., 2015	IS	57	45	100%	62	Hospital	IIEF	78.9%
Abzahandadze et al., 2017	IS and ICH	248	66	100%	64	Hospital	LSCL	26.6%
Oyewole et al., 2017	IS and ICH	121	105	100%	62	Hospital	Interview	86.8%
Winder et al., 2017	IS	52	32	100%	58	Hospital	IIEF	61.5%
Winder et al., 2017	IS	52	40	100%	61	Hospital	IIEF	76.9%
Yilmaz et al., 2017	IS	112	68	N/A	43	Hospital	Interview	60.7%
Montalvan et al., 2020	IS and ICH	150	89	100%	62	Hospital	IIEF	59.3%

© 2025 Ozkan H et al. *JAMA Network Open*.

eTable 4. Quality assessment assessed using the modified Newcastle-Ottawa scale

Variable	Study ID	Selection			Comparability	Outcome		Total (7⋆)
**Anxiety**		Representativeness of exposed cohort (⋆)	Selection of non-exposed cohort (⋆)	Ascertainment of exposure (⋆)	(⋆⋆)	Assessment of outcome (⋆)	Adequacy of follow up (⋆)	
	Langhorne et al.,2000	⋆	⋆	-	⋆	-	⋆	⋆⋆⋆⋆ (4)
	Stone et al.,2004	⋆	⋆	-	-	⋆	-	⋆⋆⋆ (3)
	Barker-Collo., 2007	⋆	⋆	⋆	⋆⋆	⋆	⋆	⋆⋆⋆⋆⋆ (6)
	Winward et al., 2009	⋆	-	-	⋆	-	-	⋆⋆ (2)
	Burton et al., 2010	⋆	⋆	-	⋆	⋆	⋆	⋆⋆⋆⋆⋆ (5)
	Skolarus et al., 2013	⋆	⋆	⋆	⋆⋆	⋆	⋆	⋆⋆⋆⋆⋆⋆⋆ (7)
	Ayerbe et al., 2014	-	-	-	⋆	⋆	-	⋆⋆ (2)
	Arba et al., 2016	⋆	⋆	⋆	⋆⋆	⋆	⋆	⋆⋆⋆⋆⋆⋆⋆ (7)
	Elf et al., 2016	⋆	-	⋆	⋆	⋆	⋆	⋆⋆⋆⋆⋆ (5)
	Maaijwee et al., 2016	⋆	⋆	⋆	⋆⋆	⋆	⋆	⋆⋆⋆⋆⋆⋆⋆ (7)
	Morris et al., 2017	⋆	⋆	⋆	⋆⋆	⋆	⋆	⋆⋆⋆⋆⋆⋆⋆ (7)
	Ojagbemi et al., 2017	⋆	-	-	⋆	⋆	⋆	⋆⋆⋆⋆ (4)
	Stein et al., 2018	⋆	⋆	-	⋆	⋆	-	⋆⋆⋆⋆ (4)
	de Graaf et al., 2018	-	⋆	-	⋆	⋆	-	⋆⋆⋆ (3)
	Chun et al., 2018	⋆	⋆	⋆	-	⋆	⋆	⋆⋆⋆⋆⋆ (5)

© 2025 Ozkan H et al. *JAMA Network Open*.

	Bovim et al., 2019	⋆	-	⋆	-	⋆	-	⋆⋆⋆ (3)
	Kapoor et al., 2019	⋆	⋆	⋆	⋆⋆	⋆	⋆	⋆⋆⋆⋆⋆⋆⋆ (7)
	Sallinen et al.,2019	⋆	-	⋆	⋆	-	-	⋆⋆⋆ (3)
	Xiao et al., 2019	⋆	⋆	-	⋆⋆	⋆	⋆	⋆⋆⋆⋆⋆⋆ (6)
	Ahmed et al., 2020	⋆	-	-	⋆	-	-	⋆⋆ (2)
	Hama et al., 2020	⋆	⋆	⋆	⋆⋆	-	⋆	⋆⋆⋆⋆⋆⋆ (6)
	Hejazi-Shirmard et al., 2020	-	-	⋆	⋆	⋆	-	⋆⋆⋆ (3)
	Rimmele et al., 2020	⋆	⋆	-	⋆	-	⋆	⋆⋆⋆⋆ (4)
	Slenders et al., 2020	⋆	⋆	-	⋆⋆	⋆	⋆	⋆⋆⋆⋆⋆⋆ (6)
	Wang et al., 2020	⋆	⋆	⋆	⋆⋆	⋆	⋆	⋆⋆⋆⋆⋆⋆⋆ (7)
	Dollenberg et al., 2021	-	⋆	-	⋆	-	⋆	⋆⋆⋆ (3)
	Wang et al., 2021	⋆	⋆	-	⋆	⋆	-	⋆⋆⋆⋆ (4)
	Ignacio et al., 2021	-	⋆	-	⋆	⋆	-	⋆⋆⋆ (3)
	Sagen-Vik et al., 2022	⋆	⋆	⋆	⋆	⋆	⋆	⋆⋆⋆⋆⋆⋆ (6)
	Redmond et al., 2022	⋆	⋆	⋆	⋆⋆	⋆	⋆	⋆⋆⋆⋆⋆⋆⋆ (7)
	Ayasrah et al., 2022	⋆	⋆	-	⋆	⋆	-	⋆⋆⋆⋆ (4)
**Depression**								

© 2025 Ozkan H et al. *JAMA Network Open*.

	Kauhanen et al., 2000	⋆	-	⋆	⋆	-	-	⋆⋆⋆ (3)
	Leppavuori et al.,2003	⋆	-	⋆	⋆⋆	⋆	⋆	⋆⋆⋆⋆⋆ (6)
	Naees et al., 2005	⋆	⋆	-	⋆	⋆	-	⋆⋆⋆⋆ (4)
	Brodaty et al., 2005	⋆	⋆	⋆	⋆	-	⋆	⋆⋆⋆⋆⋆ (5)
	Masada et al., 2007	⋆	⋆	-	⋆	⋆	-	⋆⋆⋆⋆ (4)
	Christen et al., 2009	⋆	⋆	-	⋆	⋆	⋆	⋆⋆⋆⋆⋆ (5)
	Christensen et al., 2009	⋆	⋆	-	⋆⋆	⋆	⋆	⋆⋆⋆⋆⋆⋆ (6)
	Farner et al., 2010	⋆	⋆	⋆	⋆⋆	⋆	⋆	⋆⋆⋆⋆⋆⋆⋆ (7)
	Rush et al., 2010	-	⋆	-	⋆	-	⋆	⋆⋆⋆ (3)
	White et al.,2011	-	-	⋆	⋆	⋆	-	⋆⋆⋆ (3)
	Altieri et al., 2012	-	⋆	-	⋆	-	⋆	⋆⋆⋆ (3)
	Ormstad et al., 2012	⋆	-	-	-	⋆	-	⋆⋆ (2)
	Kauranen et al., 2012	⋆	-	⋆	⋆	-	⋆	⋆⋆⋆⋆ (4)
	Naess et al., 2012	⋆	-	⋆	⋆	-	⋆	⋆⋆⋆⋆ (4)
	Ku et al., 2013	-	⋆	-	⋆	-	⋆	⋆⋆⋆ (3)
	Sibolt et al., 2013	⋆	⋆	⋆	⋆⋆	⋆	⋆	⋆⋆⋆⋆⋆⋆ (7)
	Tang et al., 2013	⋆	⋆	⋆	⋆⋆	⋆	⋆	⋆⋆⋆⋆⋆⋆⋆ (7)

© 2025 Ozkan H et al. *JAMA Network Open*.

	Murakami et al., 2013	-	⋆	-	⋆	⋆	⋆	⋆⋆⋆⋆ (4)
	Shi et al., 2014	⋆	⋆	⋆	⋆	-	-	⋆⋆⋆⋆⋆ (5)
	Koivunen et al., 2015	⋆	⋆	⋆	⋆⋆	⋆	⋆	⋆⋆⋆⋆⋆⋆⋆ (7)
	Matsuzaki et al., 2015	-	-	⋆	-	-	-	⋆ (1)
	Shi et al., 2015	-	-	⋆	⋆	-	-	⋆⋆ (2)
	Shi et al., 2015	-	⋆	-	⋆	⋆	-	⋆⋆⋆ (3)
	de Mello et al., 2016	⋆	⋆	⋆	⋆⋆	⋆	⋆	⋆⋆⋆⋆⋆⋆⋆ (7)
	Volz et al., 2016	⋆	⋆	⋆	⋆⋆	⋆	⋆	⋆⋆⋆⋆⋆⋆⋆ (7)
	Nakase et al., 2016	-	-	-	⋆	-	⋆	⋆⋆ (2)
	Maaijwee et al.,2016	⋆	⋆	⋆	⋆⋆	⋆	⋆	⋆⋆⋆⋆⋆⋆⋆ (7)
	Kootker et al.,2016	-	-	⋆	⋆	-	⋆	⋆⋆⋆ (3)
	Hirata et al., 2016	-	-	-	⋆	-	⋆	⋆⋆ (2)
	McCartney et al., 2016	-	⋆	⋆	-	⋆	⋆	⋆⋆⋆⋆ (4)
	Guiraud et al., 2016	⋆	-	⋆	-	⋆	-	⋆⋆⋆ (3)
	Cumming et al., 2016	⋆	⋆	⋆	⋆⋆	⋆	⋆	⋆⋆⋆⋆⋆⋆ (7)
	Arba et al., 2016	⋆	-	⋆	-	⋆	-	⋆⋆⋆ (3)
	Husseini et al., 2017	⋆	⋆	-	⋆	⋆	⋆	⋆⋆⋆⋆ (4)

© 2025 Ozkan H et al. *JAMA Network Open*.

	Salinas et al.,2017	⋆	⋆	⋆	⋆⋆	⋆	-	⋆⋆⋆⋆⋆⋆ (6)
	Shankar et al., 2017	⋆	⋆	⋆	⋆⋆	⋆	⋆	⋆⋆⋆⋆⋆⋆⋆ (7)
	Tessier et al., 2017	-	⋆	-	⋆	⋆	-	⋆⋆⋆ (3)
	Liang et al., 2018	⋆	⋆	⋆	-	-	⋆	⋆⋆⋆⋆ (4)
	Francis et al., 2018	⋆	⋆	⋆	⋆⋆	⋆	⋆	⋆⋆⋆⋆⋆⋆⋆ (7)
	Rimmele et al., 2020	⋆	⋆	⋆	-	-	⋆	⋆⋆⋆⋆ (4)
	Stockman-Meiland et al., 2020	-	⋆	-	⋆	⋆	-	⋆⋆⋆ (3)
	Williams and Deyemere et al., 2021	⋆	⋆	⋆	⋆⋆	⋆	⋆	⋆⋆⋆⋆⋆⋆⋆ (7)
	Ignacio et al., 2021	-	⋆	-	⋆	⋆	-	⋆⋆⋆ (3)
	Isuru et al., 2021	⋆	⋆	⋆	-	-	⋆	⋆⋆⋆⋆ (4)
	Lee et al., 2021	⋆	⋆	⋆	⋆	⋆	-	⋆⋆⋆⋆⋆ (5)
	Avadhani et al., 2021	⋆	-	⋆	⋆	-	-	⋆⋆⋆⋆⋆ (5)
	Dong et al., 2021	⋆	⋆	⋆	-	-	⋆	⋆⋆⋆⋆ (4)
	Beker et al., 2021	-	-	-	⋆	⋆	-	⋆⋆ (2)
	Ladwing et al., 2021	⋆	-	⋆	⋆	-	⋆⋆	⋆⋆⋆⋆⋆ (5)
	Sagen-Vik et al., 2022	⋆	⋆	⋆	-	-	⋆	⋆⋆⋆⋆ (4)
	Abuadas et al., 2023	-	⋆	-	⋆	⋆	-	⋆⋆⋆ (3)

© 2025 Ozkan H et al. *JAMA Network Open*.

	Murohy et al., 2023 (INTERNSTROKE)	★	★	★	★★	★	★	★★★★★★★ (7)
**Fatigue**								
	van der Werf et al., 2001	-	★	★	★	-	★	★★★★ (4)
	Dam H., 2001	-	★	-	★	★	★	★★★★ (4)
	Glander et al., 2002	-	-	★	★	-	-	★★ (2)
	Naess et al., 2005	★	★	-	★	★	★	★★★★★ (5)
	Choi-Kwon et al., 2005	★	-	★	★★	★	★	★★★★★★ (6)
	Naess et al., 2006	★	★	★	★	★	★	★★★★★★ (6)
	Schepers et al., 2006	★	★	★	★	-	★	★★★★★★ (6)
	Appelros P., 2006	★	★	★	★★	★	★	★★★★★★ (7)
	Michael et al., 2006	★	★	★	★★	★	★	★★★★★★ (7)
	Skaner et al., 2007	★	★	★	★★	★	★	★★★★★★ (7)
	Van de Port et al. 2007	★	★	★	★★	★	★	★★★★★★ (7)
	Christensen et al., 2008	★	★	★	★★	★	★	★★★★★★ (7)
	Park et al., 2009	-	-	★	-	★	-	★★ (2)
	Windward et al., 2009	-	-	★	-	★	★	★★★ (3)

© 2025 Ozkan H et al. *JAMA Network Open*.

	Snaphaan L, Werf S.v.d, Leeuw., 2010	-	⋆	⋆	-	⋆	-	⋆⋆⋆ (3)
	Tang et al., 2010	⋆	-	-	⋆	⋆	⋆	⋆⋆⋆⋆ (4)
	Mead et al., 2011	⋆	⋆	⋆	⋆⋆	⋆	⋆	⋆⋆⋆⋆⋆⋆⋆ (7)
	Lerdal A, and Kottorp A., 2011	⋆	⋆	⋆	⋆⋆	⋆	⋆	⋆⋆⋆⋆⋆⋆⋆ (7)
	Jerath et al., 2011	⋆	⋆	-	⋆	-	-	⋆⋆⋆ (3)
	Chesnut T.J., 2011	-	-	⋆	-	⋆	-	⋆⋆ (2)
	Crosby et al., 2012	⋆	⋆	⋆	⋆	⋆	⋆	⋆⋆⋆⋆⋆⋆ (6)
	Hubacher et al.,2012	-	⋆	-	⋆⋆	⋆	-	⋆⋆⋆⋆ (4)
	Parks et al., 2012	⋆	⋆	⋆	⋆	⋆	⋆	⋆⋆⋆⋆⋆⋆ (6)
	Radman et al., 2012	⋆	⋆	⋆	⋆⋆	⋆	-	⋆⋆⋆⋆⋆⋆ (6)
	Tang et al., 2012	⋆	⋆	⋆	⋆	⋆	⋆	⋆⋆⋆⋆⋆⋆ (6)
	Wu et al., 2013	⋆	-	⋆	⋆	-	-	⋆⋆⋆⋆⋆ (5)
	Miller et al., 2013	-	⋆	-	⋆	-	⋆	⋆⋆⋆ (3)
	Tang et al., 2014	⋆	⋆	-	⋆	-	⋆	⋆⋆⋆ (3)
	Maaijwee et al., 2014	⋆	-	⋆	-	⋆	-	⋆⋆⋆ (3)
	Carlsson G.E., 2015	-	-	-	⋆	⋆	-	⋆⋆ (2)
	Duncan et al., 2015	⋆	⋆	-	⋆⋆	-	⋆	⋆⋆⋆⋆ (4)
	Ponchel et al., 2016	⋆	⋆	⋆	⋆	⋆	⋆	⋆⋆⋆⋆⋆⋆ (6)

© 2025 Ozkan H et al. *JAMA Network Open*.

	Drummond et al., 2017 (NotFAST)	⋆	⋆	⋆	⋆	⋆	⋆	⋆⋆⋆⋆⋆⋆ (6)
	Hawkins et al., 2017	⋆	-	⋆	⋆	-	⋆	⋆⋆⋆⋆ (4)
	Mutai et al., 2017	⋆	-	⋆	⋆⋆	⋆	-	⋆⋆⋆⋆⋆ (5)
	Sarfo et al., 2018	⋆	⋆	⋆	⋆	⋆	⋆	⋆⋆⋆⋆⋆⋆ (6)
	Wang et al., 2018	⋆	⋆	⋆	-	-	⋆	⋆⋆⋆⋆ (4)
	Morsund et al., 2018	⋆	⋆	⋆	⋆⋆	⋆	⋆	⋆⋆⋆⋆⋆⋆⋆ (7)
	Katzan et al., 2019	⋆	⋆	⋆	⋆⋆	⋆	⋆	⋆⋆⋆⋆⋆⋆⋆ (7)
	Kjeverud et al., 2020	⋆	-	⋆	⋆⋆	⋆	-	⋆⋆⋆⋆⋆⋆ (6)
	Su et al., 2020	⋆	⋆	-	⋆	⋆	⋆	⋆⋆⋆⋆⋆⋆ (6)
	Almhdawi et al., 2021	⋆	⋆	⋆	⋆	⋆	-	⋆⋆⋆⋆⋆⋆ (6)
	Ulrichsen et al., 2021	⋆⋆	-	⋆	⋆⋆	-	⋆	⋆⋆⋆⋆⋆⋆ (6)
	Vituri et al., 2021	-	-	⋆	⋆	⋆	-	⋆⋆⋆ (3)
	Pedersen et al., 2022	⋆	⋆	-	⋆	⋆	⋆	⋆⋆⋆⋆⋆ (5)
	Schnitzer et al., 2023	⋆	⋆⋆	-	⋆⋆	⋆	⋆	⋆⋆⋆⋆⋆⋆⋆ (7)
**Sleep Disturbance**								
	Parra et al., 1999	-	-	⋆	-	-	-	⋆ (1)
	Wessendorf et al., 2000	⋆	⋆	⋆	⋆⋆	⋆	⋆	⋆⋆⋆⋆⋆⋆⋆ (7)

© 2025 Ozkan H et al. *JAMA Network Open*.

	Szucs et al., 2002	-	⋆	-	⋆	-	-	⋆⋆ (2)
	Iranzo et L., 2002	-	-	⋆	⋆	⋆	-	⋆⋆⋆ (3)
	Leppävuori et al., 2002	⋆	⋆	⋆	⋆	⋆	⋆	⋆⋆⋆⋆⋆⋆ (6)
	Nachtman et al., 2003	⋆	-	-	-	-	⋆	⋆⋆ (2)
	Martinez et al., 2005	⋆	⋆	⋆	⋆	⋆	-	⋆⋆⋆⋆⋆ (5)
	Huhtakangas et al., 2005	⋆	⋆	⋆	-	⋆	⋆	⋆⋆⋆⋆⋆ (5)
	Bassetti et al., 2006	⋆	⋆	-	⋆	-	⋆	⋆⋆⋆⋆ (4)
	C.L., Milanova M., and Gugger M., (2006)	⋆	-	⋆	⋆	⋆	-	⋆⋆⋆⋆ (4)
	Dziewas et al., 2007	⋆	⋆	-	⋆	-	⋆	⋆⋆⋆⋆ (4)
	Koch et al., 2007	⋆	⋆	⋆	⋆⋆	⋆	⋆	⋆⋆⋆⋆⋆⋆⋆ (7)
	Leino et al., 2010	-	-	⋆	⋆	-	-	⋆⋆ (2)
	Mansukhani et al., 2010	⋆	⋆	-	⋆	⋆	⋆	⋆⋆⋆⋆⋆ (5)
	Chen et al., 2011	-	-	⋆	-	⋆	-	⋆⋆ (2)
	Hsieh et al., 2012	-	⋆	⋆	-	⋆	-	⋆⋆⋆ (3)
	Naess H., Lunde L., Brogger J., 2012	⋆	-	⋆	-	⋆	-	⋆⋆⋆ (3)
	Suh et al., 2013	⋆	⋆	⋆	⋆⋆	⋆	⋆	⋆⋆⋆⋆⋆⋆⋆ (7)
	Brown et al., 2013	-	-	-	⋆	-	-	⋆ (1)

© 2025 Ozkan H et al. *JAMA Network Open*.

	Tang et al., 2014	⋆	⋆	-	⋆	⋆	⋆	⋆⋆⋆⋆⋆ (5)
	Zhang et al., 2014	⋆	⋆	⋆	⋆⋆	⋆	⋆	⋆⋆⋆⋆⋆⋆⋆ (7)
	Aaronson et al., 2015	⋆	-	⋆	⋆	-	-	⋆⋆⋆ (3)
	Chen et al., 2015	-	⋆	-	⋆	⋆	⋆	⋆⋆⋆⋆ (4)
	Lipford et al., 2015	-	-	⋆	-	⋆	-	⋆⋆ (2)
	Chen et al., 2015	⋆	⋆	-	⋆	⋆	⋆	⋆⋆⋆⋆⋆ (5)
	Gupta et al., 2015	⋆	-	⋆	⋆	-	-	⋆⋆⋆ (3)
	Lisabeth et al., 2016 (BASIC STUDY)	⋆	⋆	⋆	⋆⋆	⋆	⋆	⋆⋆⋆⋆⋆⋆⋆ (7)
	Koo et al., 2016	-	⋆	⋆	⋆	-	⋆	⋆⋆⋆⋆ (4)
	Camilo et al.,2016	⋆	⋆	⋆	⋆	⋆	⋆	⋆⋆⋆⋆⋆⋆ (6)
	Bravata et al., 2017	-	-	-	⋆	⋆	-	⋆⋆ (2)
	Menon, Sukumaran, Varma, and Radhakrishnan., 2017	⋆	⋆	⋆	-	⋆	-	⋆⋆⋆⋆ (4)
	Huhtakangas et al., 2017	⋆	⋆	⋆	⋆	⋆	⋆	⋆⋆⋆⋆⋆⋆ (6)
	Scherbakov et al., 2017	⋆	⋆	⋆	⋆	⋆	⋆	⋆⋆⋆⋆⋆⋆ (6)
	Kumar, Suri, and Manoca., 2017	⋆	⋆	⋆	-	⋆	-	⋆⋆⋆⋆ (4)
	Fisse et al., 2017	⋆	-	⋆	⋆	⋆	⋆	⋆⋆⋆⋆⋆ (5)

© 2025 Ozkan H et al. *JAMA Network Open*.

	Kim et al., 2017	⋆	⋆	⋆	-	⋆	-	⋆⋆⋆⋆ (4)
	Kim et al., 2017	-	⋆	-	⋆	⋆	-	⋆⋆⋆ (3)
	Slonkova et al., 2017	⋆	-	-	-	⋆	⋆	⋆⋆⋆ (3)
	Gadodia et al., 2018	-	-	⋆	-	-	-	⋆⋆ (2)
	Boulos et al., 2018	⋆	⋆	⋆	⋆⋆	⋆	⋆	⋆⋆⋆⋆⋆⋆⋆ (7)
	Festic et al., 2018	⋆	-	-	⋆	-	⋆	⋆⋆⋆⋆ (4)
	Zhang et al., 2019	⋆	⋆	⋆	⋆⋆	-	⋆	⋆⋆⋆⋆⋆⋆ (6)
	Haba-Rubio et al., 2019	-	⋆	-	⋆	-	⋆	⋆⋆⋆ (3)
	Padmaja, Krishnaswamy, and Javali., 2019	⋆	⋆	⋆	⋆⋆	⋆	⋆	⋆⋆⋆⋆⋆⋆⋆ (7)
	Nair et al., 2019	⋆	⋆	-	⋆	-	⋆	⋆⋆⋆ (3)
	Lisabeth et al., 2019 (Corpus Christi Project)	⋆	⋆	⋆	⋆⋆	⋆	⋆	⋆⋆⋆⋆⋆⋆⋆ (7)
	Brown et al., 2019 (BASIC Study)	⋆	⋆	⋆	⋆	⋆	⋆	⋆⋆⋆⋆⋆⋆ (6)
	Mohammad et al., 2019	⋆	-	⋆	-	⋆	-	⋆⋆⋆ (3)
	Dharmakulaseelan et al., 2020	⋆	⋆	-	⋆	⋆	⋆	⋆⋆⋆⋆⋆ (5)
	Barretto et al., 2020	⋆	⋆	-	⋆	⋆	⋆	⋆⋆⋆⋆⋆ (5)

© 2025 Ozkan H et al. *JAMA Network Open*.

	Sekplin et al., 2020	-	-	⋆	-	⋆	⋆	⋆⋆⋆⋆ (4)
	Katzan et al.,2020	⋆	⋆	⋆	⋆	⋆	⋆	⋆⋆⋆⋆⋆⋆⋆ (7)
	Ho et al., 2021	-	-	⋆	-	⋆	⋆	⋆⋆⋆ (3)
**Social Participation**								
	Mair et al., 2006	⋆	⋆	⋆	⋆	⋆	⋆	⋆⋆⋆⋆⋆⋆ (6)
	Sit et al., 2007	⋆	⋆	⋆	⋆⋆	⋆	⋆	⋆⋆⋆⋆⋆⋆⋆ (7)
	Shyu et al., 2008	⋆	⋆	⋆	⋆⋆	⋆	-	⋆⋆⋆⋆⋆⋆ (6)
	Muraki et al., 2008	⋆	⋆	-	⋆⋆	-	⋆	⋆⋆⋆⋆⋆ (5)
	Achten et al., 2012	-	⋆	-	⋆	-	-	⋆⋆ (2)
	Petersen et al., 2012	⋆	⋆	-	⋆⋆	⋆	⋆	⋆⋆⋆⋆⋆⋆ (6)
	Sreedharan et al., 2013	⋆	⋆	⋆	⋆⋆	⋆	⋆	⋆⋆⋆⋆⋆⋆⋆ (7)
	Francois et al., 2013	-	⋆	⋆	⋆⋆	⋆	-	⋆⋆⋆⋆⋆ (5)
	Yu et al., 2013	⋆	⋆	⋆	⋆	⋆	⋆	⋆⋆⋆⋆⋆⋆ (6)
	Baumann et al., 2014	⋆	⋆	⋆	⋆⋆	⋆	⋆	⋆⋆⋆⋆⋆⋆⋆ (7)
	Jaracz et al., 2015	⋆	⋆	-	⋆	⋆	-	⋆⋆⋆⋆ (4)
	Vincent-Onabajo et al., 2016	⋆	-	⋆	⋆⋆	-	⋆	⋆⋆⋆⋆⋆ (5)
	Wolf et al., 2016	⋆	-	⋆	⋆	⋆	-	⋆⋆⋆⋆ (4)
	Chen et al., 2016	⋆	⋆	⋆	⋆⋆	⋆	⋆	⋆⋆⋆⋆⋆⋆⋆ (7)

© 2025 Ozkan H et al. *JAMA Network Open*.

	Tse et al., 2017	⋆	⋆	⋆	-	⋆	⋆	⋆⋆⋆⋆⋆ (5)
	Liu et al., 2019	⋆	⋆	-	⋆⋆	⋆	⋆	⋆⋆⋆⋆⋆⋆ (6)
	Li et al., 2020	⋆	-	⋆	⋆	-	⋆	⋆⋆⋆⋆ (4)
	Li et al., 2021	⋆	⋆⋆	-	⋆	-	⋆⋆	⋆⋆⋆⋆⋆⋆ (6)
	Ekstrand and Bogardh et al., 2022	-	⋆	⋆⋆	-	⋆	⋆	⋆⋆⋆⋆⋆ (5)
**Pain**								
	Bowsher et al., 2001	-	⋆	-	⋆	⋆	-	⋆⋆⋆ (3)
	Weimar et al., 2002	-	-	⋆	-	-	⋆	⋆⋆ (2)
	Widar et al., 2002	⋆	⋆	-	⋆	⋆	⋆	⋆⋆⋆⋆⋆ (5)
	Kong et al., 2004	⋆	⋆	⋆	⋆⋆	⋆	⋆	⋆⋆⋆⋆⋆⋆⋆ (7)
	Widar et al., 2004	-	⋆	-	⋆	-	-	⋆⋆ (2)
	Jonson et al., 2006)	⋆	⋆	-	⋆	⋆	-	⋆⋆⋆⋆ (4)
	Apperlos P,.2006	-	⋆	-	⋆	-	-	⋆⋆ (2)
	Skaner et al., 2007	⋆	⋆	⋆	⋆⋆	⋆	⋆	⋆⋆⋆⋆⋆⋆⋆ (7)
	Sackley et al., 2008	-	⋆	-	⋆	⋆	⋆	⋆⋆⋆⋆ (4)
	Indredavik et al., 2008	⋆	-	⋆	-	-	⋆	⋆⋆⋆ (3)
	Ludstrom et al., 2009	-	⋆	⋆	-	⋆	-	⋆⋆⋆ (3)
	Klit et al., 2011	⋆	⋆	⋆	⋆	⋆	⋆	⋆⋆⋆⋆⋆⋆ (6)

© 2025 Ozkan H et al. *JAMA Network Open*.

	Sprigg et al., 2011	⋆	-	-	⋆	-	⋆	⋆⋆⋆ (3)
	de Oliveiraet al., 2012	-	⋆	-	-	⋆	-	⋆⋆ (2)
	Naess et al., 2012	⋆	-	⋆	⋆	-	⋆	⋆⋆⋆⋆ (4)
	Hansen et al., 2012	⋆	⋆	⋆	⋆⋆	-	⋆	⋆⋆⋆⋆⋆⋆ (6)
	Gamble et al., 2012	⋆	⋆	⋆	⋆⋆	⋆	⋆	⋆⋆⋆⋆⋆⋆⋆ (7)
	Kuptniratsaikul et al., 2013	-	⋆	-	⋆	⋆	-	⋆⋆⋆ (3)
	O'Donnell et al., 2013	⋆	⋆	-	⋆	⋆	⋆	⋆⋆⋆⋆⋆ (5)
	Raffaeli et al., 2013	⋆	⋆	⋆	⋆⋆	⋆	⋆	⋆⋆⋆⋆⋆⋆⋆ (7)
	Kuptniratsaikul et al., 2013	⋆	⋆	-	⋆	-	-	⋆⋆⋆ (3)
	Harno et al., 2014	⋆	⋆	⋆	⋆⋆	⋆	⋆	⋆⋆⋆⋆⋆⋆⋆ (7)
	Koivunen et al., 2015	⋆	⋆	⋆	⋆⋆	⋆	⋆	⋆⋆⋆⋆⋆⋆⋆ (7)
	Paolluci et al., 2016	⋆	⋆	-	⋆	⋆	-	⋆⋆⋆⋆ (4)
	Choi-Kwon et al., 2016	⋆	-	⋆	⋆⋆	-	⋆	⋆⋆⋆⋆ (5)
	Paolluci et al., 2016	⋆	⋆	⋆	⋆⋆	⋆	⋆	⋆⋆⋆⋆⋆⋆⋆ (7)
	Bashir et al., 2017	⋆	-	⋆	-	⋆	-	⋆⋆⋆ (3)
	Osama et al., 2018	⋆	⋆	⋆	⋆⋆	⋆	⋆	⋆⋆⋆⋆⋆⋆⋆ (7)

© 2025 Ozkan H et al. *JAMA Network Open*.

	Kim et al., 2018	⋆	-	-	⋆	⋆	⋆	⋆⋆⋆⋆ (4)
	Vukojevic et al., 2018	-	⋆	⋆	⋆	-	⋆	⋆⋆⋆⋆ (4)
	Katzan et al., 2018	⋆	⋆	⋆	⋆	-	-	⋆⋆⋆⋆ (4)
	Katzan et al., 2018	⋆	-	⋆	-	⋆	⋆	⋆⋆⋆ (4)
	Lindgren et al., 2018	-	⋆	⋆	-	⋆	-	⋆⋆⋆ (3)
	Westerlind et al., 2020	⋆	⋆	⋆	⋆⋆	⋆	⋆	⋆⋆⋆⋆ (4)
	Mhangara et al., 2020	⋆	-	⋆	-	-	-	⋆⋆ (2)
**Constipation**								
	Robain et al., 2002	⋆	⋆	⋆	⋆	⋆	⋆	⋆⋆⋆⋆⋆⋆ (6)
	Harari et al., 2002	⋆	⋆	⋆	⋆	⋆	⋆	⋆⋆⋆⋆⋆⋆ (6)
	Baztan et al., 2003	⋆	-	⋆	⋆	-	⋆	⋆⋆⋆⋆ (4)
	Harari et al., 2004	⋆	⋆	⋆	⋆⋆	⋆	⋆	⋆⋆⋆⋆⋆⋆⋆ (7)
	Otegbayo et al., 2006	-	-	⋆	⋆	-	⋆	⋆⋆⋆ (3)
	Su et al., 2009	⋆	-	⋆	-	⋆	-	⋆⋆⋆ (3)
	Dourado et al., 2012	-	⋆	-	⋆	-	-	⋆⋆ (2)
	Sun et al., 2022	⋆	⋆⋆	-	-	⋆⋆	⋆	⋆⋆⋆⋆⋆⋆ (6)
**Faecal incontinence**								
	Harari et al., 2002	⋆	⋆	-	⋆	-	⋆	⋆⋆⋆⋆ (4)

© 2025 Ozkan H et al. *JAMA Network Open*.

	Brittain et al., 2006	⋆	-	⋆	-	⋆	-	⋆⋆⋆ (3)
	Jacop L., and Kostev K., 2019	-	⋆	-	⋆	-	⋆	⋆⋆⋆ (3)
**Bladder Dysfunction**								
	van Kujik et al., 2001	⋆	-	⋆	-	⋆	⋆	⋆⋆⋆⋆ (4)
	Patel et al., 2001	-	⋆	⋆	-	⋆	⋆	⋆⋆⋆⋆ (4)
	Lawrance et al., 2001	⋆	-	⋆	-	⋆	-	⋆⋆⋆ (3)
	Kolominsky-Rabas et al., 2003	⋆	⋆	-	-	⋆	-	⋆⋆⋆ (3)
	Jorgensen et al., 2004	-	⋆	⋆	⋆	-	⋆	⋆⋆⋆⋆ (4)
	McLaren et al., 2005	⋆	⋆	-	⋆	⋆	-	⋆⋆⋆⋆ (4)
	Pettersen et al., 2007	⋆	-	⋆	-	⋆	-	⋆⋆⋆ (3)
	Tibaek et al., 2008	⋆	⋆	⋆	⋆⋆	⋆	⋆	⋆⋆⋆⋆⋆⋆⋆ (7)
	Mizrah et al., 2011	-	⋆	-	⋆	⋆	-	⋆⋆⋆ (3)
	Itoh et al., 2012	⋆	-	⋆	⋆	-	⋆	⋆⋆⋆ (3)
	Pizzi et al., 2013	-	⋆	-	⋆	⋆	-	⋆⋆⋆ (3)
	Cai et al., 2013	⋆	⋆	⋆	⋆⋆	⋆	⋆	⋆⋆⋆⋆⋆⋆⋆ (7)
	Xiong et al., 2014	⋆	⋆	-	⋆	⋆	-	⋆⋆⋆⋆ (4)
	Idiaquez et al., 2015	⋆	-	⋆	⋆	-	⋆	⋆⋆⋆⋆ (4)

© 2025 Ozkan H et al. *JAMA Network Open*.

	Woo et al., 2016	-	⋆	-	⋆	⋆	⋆	⋆⋆⋆⋆⋆⋆⋆ (7)
**Sexual dysfunction**								
	Kimura et al., 2001	⋆	-	⋆	-	⋆	⋆	⋆⋆⋆⋆ (4)
	Choi-Kwon S, and Kim J.S., 2002	⋆	-	⋆	⋆	-	⋆	⋆⋆⋆⋆ (4)
	Cheung R.t.F., 2002	-	⋆	-	-	⋆	-	⋆⋆ (2)
	Giaquinto et al., 2003	⋆	⋆	-	⋆	-	⋆	⋆⋆⋆⋆ (4)
	Jung et al., 2007	⋆	⋆	⋆	⋆	⋆	⋆	⋆⋆⋆⋆⋆⋆ (6)
	Bener et al., 2008	⋆	⋆	-	⋆	-	⋆	⋆⋆⋆⋆ (4)
	Hilz et al., 2012	⋆	⋆	⋆	⋆⋆	⋆	⋆	⋆⋆⋆⋆⋆⋆⋆ (7)
	Stein et al., 2012	-	⋆	-	⋆	-	-	⋆⋆ (2)
	Bugnicourt et al., 2014	⋆	⋆	⋆	⋆	⋆	⋆	⋆⋆⋆⋆⋆⋆ (6)
	Koehn et al., 2015	⋆	-	⋆	⋆	-	⋆	⋆⋆⋆⋆⋆ (5)
	Abzahandadze et al., 2017	-	⋆	⋆	-	⋆	-	⋆⋆⋆ (3)
	Oyewole et al., 2017	⋆	⋆	-	⋆	⋆	⋆	⋆⋆⋆⋆⋆ (5)
	Winder et al., 2017	⋆	⋆	⋆	⋆	⋆	⋆	⋆⋆⋆⋆⋆⋆ (6)
	Winder et al., 2017	⋆	⋆	⋆	⋆	-	⋆	⋆⋆⋆⋆⋆⋆ (6)
	Yilmaz et al., 2017	⋆	-	⋆	-	⋆	-	⋆⋆⋆ (3)

© 2025 Ozkan H et al. *JAMA Network Open*.

	Montalvan et al., 2020	-	⋆	-	⋆	-	⋆	⋆⋆⋆ (3)

© 2025 Ozkan H et al. *JAMA Network Open*.

eTable 5. Characteristics of the Included Studies

Variable	Studies, No. (%)										
	All[6,18,21,24,28,29,32,38-289,291-307]	Anxiety[6,18,24,28,32,38-62]	Depression[21,29,63-111]	Fatigue[21,28,66,112-152]	Sleep disturbance[153-198]	Social participation[199-216]	Pain[217-246,307]	Constipation[247-254,308]	Fecal incontinence[250,255,256]	Bladder dysfunction[257-273]	Sexual dysfunction[274-289]
No. of studies	279	32	52	48	50	20	32	8	3	17	17
No. of participant	117 440	2277	34 672	13 153	10 228	4056	25 448	1057	18 419	6053	2077
Age, median (range)	65 (59-69)	65 (39-76)	65 (33.6-81)	62 (29-79)	65.5 (38.3-73)	67 (42-75)	66 (42-78)	65 (41-79)	67 (57-70)	71 (55-80)	61 (43-64)
Reported sex											
Proportion of studies with a dominance of female participants	70 (25)	7 (21.9)	14 (26.9)	22 (45.2)	13 (26.0)	8 (40.0)	15 (46.9)	3 (37.5)	NA	7 (41.2)	2 (11.8)
Proportion of male participants reported in the studies	209 (75)	25 (78.1)	38 (73.1)	26 (54.2)	37 (74.0)	12 (60.0)	17 (53.1)	5 (62.5)	3 (100.0)	10 (58.8)	15 (88.2)
Studies on ischemic stroke	160 (57.3)	12 (37.5)	38 (73.1)	23 (47.9)	45 (90)	12 (60)	10 (31.3)	3 (37.5)	NA	8 (47.1)	9 (52.9)
Studies on ICH	6 (2.2)	1 (3.1)	2 (3.8)	NA	NA	NA	3 (9.4)	NA	NA	NA	NA
Studies on mixed cohorts ischemic stroke and ICH	113 (40.5)	19 (59.4)	12 (23.1)	25 (52.1)	5 (10)	8 (40)	19 (59.4)	5 (62.5)	3 (100)	9 (52.9)	8 (47.1)
Follow-up time from stroke, median (range)	76.9 (1-120)	3 (1-120)	6 (1-120)	12 (1-118)	3 (1-120)	6 (1-36)	12 (3-120	9 (1-48)	6 (3-120)	3 (1-108)	3 (1-84)
Hospital-based studies	188 (67.4)	25 (78.12)	36 (69.23)	29 (60.42)	31 (62.0)	17 (85)	19 (59.38)	6 (75.0)	2 (66.67)	11 (64.1)	12 (70.59)

© 2025 Ozkan H et al. *JAMA Network Open*.

Population-based studies	91 (32.06)	7 (21.88)	16 (30.77)	19 (39.58)	19 (38.0)	3 (15)	13 (40.62)	2 (25.0)	1 (33.33)	6 (35.29)	5 (29.04)

© 2025 Ozkan H et al. *JAMA Network Open*.

eFigure 4. Natural history of nonmotor outcomes ((G Pain), (H Anxiety), I Depression), (J Fecal Incontinence)

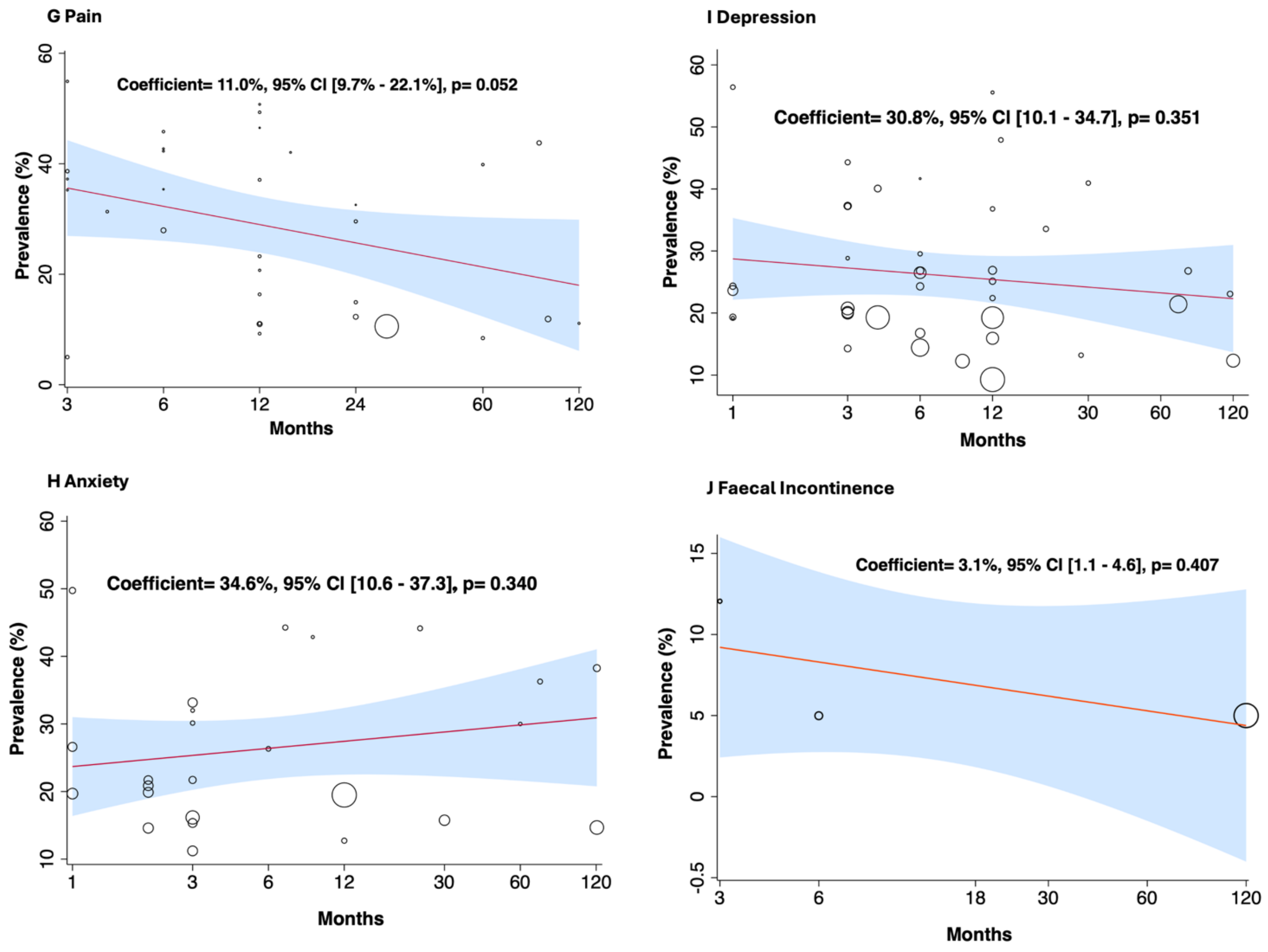

Time to follow-up adjusted meta-regression natural history graph adjusted for time to follow-up and the prevenance estimate reported in each study. The red line represents the fitted regression model, while the blue shaded area indicates the 95% confidence interval.

eAppendix. Summary of sources of heterogeneity

We assessed study heterogeneity using random effects subgroup analysis, I^2, and H statistics, where an I^2 of 75% indicated substantial heterogeneity. High heterogeneity was observed across all non-motor outcomes among studies in each domain (ranging from I^2 52.3% to 98.7%). Consequently, we investigated sources of heterogeneity through random effects subgroup analysis models. The first model incorporated study-level characteristics such as age, sex, stroke type, and outcome measure (see Table 5). The second model focused on non-motor outcomes with multiple symptoms describing the same domain, including sleep disturbance, pain, bladder dysfunction, and sexual dysfunction (see to Figure 3, 1 to 1.4).

© 2025 Ozkan H et al. *JAMA Network Open*.

The source of heterogeneity varied across each non-motor outcome domain; however, a consistent observation was that a significant portion of the heterogeneity remained unexplained by study characteristics and sub-symptomology. Sub-group analysis at the study level revealed that, for non-motor outcomes such as anxiety, depression, pain, and constipation, the stroke type could account for 10.71% to 59.82% of the observed heterogeneity (I^2). Additionally, the median age in the studies explained 37.14% to 56.43% of the heterogeneity for constipation and sexual dysfunction. Sex was found to account for I^2 values ranging from 36.37% to 74.30% for reduced social participation and constipation. Lastly, the choice of outcome measure explained 37.67% to 72.67% of the heterogeneity for anxiety, sleep disturbance, pain, constipation, and sexual dysfunction.

For non-motor outcomes that is presented with sub-symptoms, the sleep disturbance (see figure 2, 1.0), studies that qualified for sleep disturbance described 3 common types of sleep related problems after stroke including (figure 2, 1.0): obstructive sleep apnoea; sleep-disordered breathing; and sleep apnoea/insomnia. Between study heterogeneity was significantly high in studies reporting obstructive sleep apnoea (I^2 90.51%), and sleep apnoea/insomnia (I^2 97.40%), but was significantly low for sleep-disordered breathing (I^2 0.06%, p= 0.07). We identified that compared with obstructive sleep apnoea, sleep apnoea/insomnia subtypes, the group of studies in the sleep-disordered breathing had the highest prevalence 63% (range 57% – 73%), *vs* 57% (range 19% – 73%), 58% (range 23% –77%) respectively. In comparison to other sub-groups of sleep disturbance, sleep-disordered breathing group commonly included large cohort population-based ischaemic stroke studies (Lisabeth et al., 2016 –BASIC Study; Lisabeth et al., 2019 – Corpus Christi Project; and Brown et al., 2019) with a median follow-up up to 12 months after stroke.

For pain domain (see figure 2, 1.1), studies that qualified for pain domain described 3 common types of post stroke pain including: unexplained pain; central post stroke pain (CPSP); bodily pain; and unexplained pain. Between study heterogeneity was significantly high in studies reporting unexplained pain (I^2= 98.07%), unexplained bodily pain (*I^2= 98.07%),* CPSP (I^2= *97.21%*), then unexplained shoulder pain (*I^2= 94.84%).* Unexplained pain type included 4 registry data with different ethnic groups, study cohort size, screening tools, stroke type, and age groups this may be the reason for high level of between study heterogeneity. In CPSP two studies Osama et al., 2018 and Kim et al., 2018 reported higher prevalence of pain 35% - 47% than other 7 studies. In unexplained pain 4 studies reported a lower prevenance rate between 11% - 21% compared with 12 other studies (31% - 55%)

© 2025 Ozkan H et al. *JAMA Network Open*.

investigating unexplained pain type. Lastly one study in shoulder pain domain Gamle et al., 2012 reported higher prevalence 42% than other 2 studies.

For bladder problems (see figure 2, 1.2), subgroup analysis stratified by different balder problems after stroke, there was little or no significant improvement in rate of between study heterogeneity (urinary incontinence I^2= 98.16% *vs* urinary urgency $I^{2=}$ 95.24%). In urinary incontinence domain, the commonality between studies reporting high (45% -79%) prevalence rate including: Pizzi et al., 2013; Tibaek et al., 2008; Kolominsky-Rabas et al., 2003; and Mizrah et al., 2011 commonly used Barthel Index scale as a measure of bladder function, whereas other studies reporting low prevalence used mixture of methods to measure bladder function including nurse led interview to Modified Barthel ADL Index scale.

For sexual dysfunction domain (see figure 2, 1.3), there were no significant differences in rate of heterogeneity after the subgroup analysis between two (I^2= 94.38% vs I^2= 91.63%) different domains of sexual dysfunction after stroke. In both groups the prevalence rate of sexual dysfunction after stroke were high (32% to 87%), except for one study Abzahandadze et al., 2017 reporting prevalence rate if 27%, which investigated erectile dysfunction and relationship between stroke survivors and spouses. Except for bias towards screening erectile dysfunction in men, and differences in screening tools to measure sexuality after stroke there were no other reasons for source of high heterogeneity rate.

© 2025 Ozkan H et al. *JAMA Network Open*.

eTable 6. Adjusted study-level characteristics associated with the prevalence of adverse nonmotor outcomes

**Variables**	**Non-Motor Outcomes**<br><br>**OR [95% CI], *P* Value**									
	Anxiety	Depression	Fatigue	Sleep Disturbance	Social Participation	Pain	Constipation	Faecal Incontinence	Bladder Dysfunction	Sexual Dysfunction
Mixed case stroke type (Reference: Ischaemic)	**2·06 [1·41 – 3·27] 0.014**	0·92 [0·63 – 1·54] 0·588	**1·53 [1·16 – 1·75] 0·010**	0·65 [0·48 – 1·01] 0·378	**2·69 [1·67 – 3·13] 0·007**	**1·07 [1·01 – 2·63] 0·053**	**3·51 [1·70 – 4·21] 0·015**	N/A all were on ischaemic stroke	1·94 [1·13 – 2·62] 0·084	**1·51 [1·28 – 2·88] 0·032**
Age >55 years old (Reference: <54 years old)	0·98 [0·71 – 1·05] 0·133	0·40 [0·18 – 1·05] 0·120	**1·18 [1·09 – 2·32] 0·048**	0·93 [0·80 – 1·18] 0·834	**1·23 [1·06 – 1·72] 0·023**	**1·78 [1·17 – 1·91] 0·015**	0·95 [0·71 – 1·08] 0·781	**3·80 [2·42 – 4·53] 0·031**	0·71 [0·31 – 1·25] 0·453	0·82 [0·61 – 1·16] 0·562
Female sex (Reference: Male sex)	1·11 [0·93 – 1·35] 0·945	**1·82 [1·16 – 3.97] 0.034**	1·02 [0·78 – 1·39] 0·205	**1·46 [1·11 – 2·63] 0·033**	0·94 [0·80 – 1·62] 0·773	0·91 [0·79 – 1·24] 0·352	0·98 [0·87 – 1·07] 0·679	**2·31 [1·04 – 2·71] 0·023**	**1·13 [1·04 – 2·28] 0·019**	0·34 [0·16 – 1·03] 0·124
Hospital-based study design (Reference: Population)	**1·73 [1·10 – 2·17] 0·017**	**1·60 [1·11 – 1·82] 0·008**	0·89 [0·72 – 1·04] 0·212	1·17 [1·02 – 1·62] 0·054	**1·83 [1·09 – 2·06] 0·040**	0·97 [0·81 – 1·48] 0·804	0·18 [0·16 – 1·01] 0·326	**2·70 [1·03 – 3·14] <0.0001**	0·92 [0·81 – 0·98] 0·371	**1·37 [1·19 – 2·64] 0·019**

*Mixed case stroke type = studies with cohorts including both intracerebral haemorrhage and ischaemic stroke

© 2025 Ozkan H et al. *JAMA Network Open*.

eFigure 5. Sources for heterogeneity assessed using random effects meta-analysis model (subgroup-analysis of each nonmotor outcome with sub-symptoms)

1.1 Sleep Disturbance

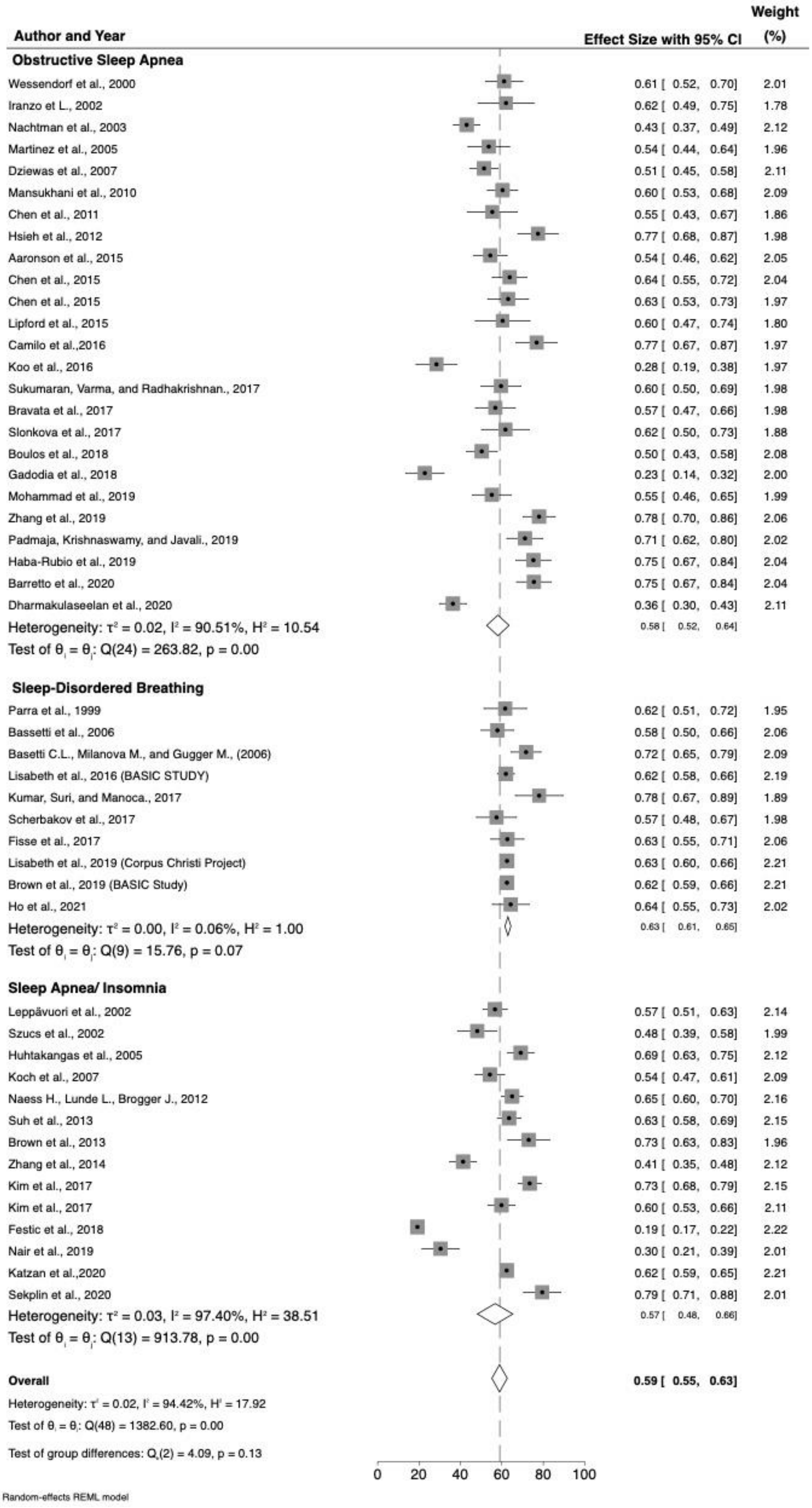

© 2025 Ozkan H et al. *JAMA Network Open*.

1.2 Pain

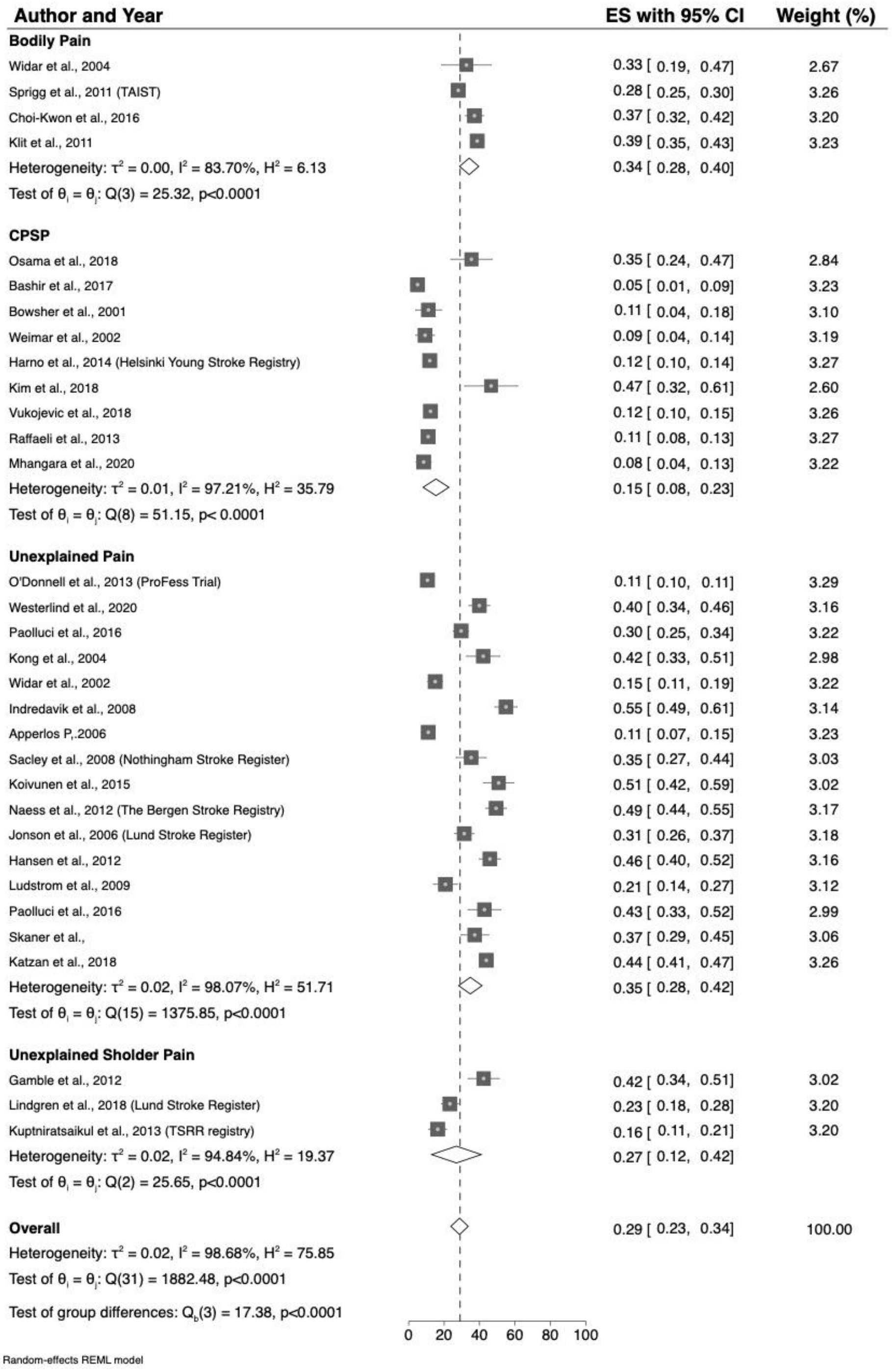
Author and Year
ES with 95% CI
Weight (%)
Bodily Pain
Widar et al., 2004 0.33 [0.19, 0.47] 2.67
Sprigg et al., 2011 (TAIST) 0.28 [0.25, 0.30] 3.26
Choi-Kwon et al., 2016 0.37 [0.32, 0.42] 3.20
Klit et al., 2011 0.39 [0.35, 0.43] 3.23
Heterogeneity: τ^2 = 0.00, I^2 = 83.70%, H^2 = 6.13 0.34 [0.28, 0.40]
Test of $\theta_i = \theta_j$: Q(3) = 25.32, p<0.0001
CPSP
Osama et al., 2018 0.35 [0.24, 0.47] 2.84
Bashir et al., 2017 0.05 [0.01, 0.09] 3.23
Bowsher et al., 2001 0.11 [0.04, 0.18] 3.10
Weimar et al., 2002 0.09 [0.04, 0.14] 3.19
Harno et al., 2014 (Helsinki Young Stroke Registry) 0.12 [0.10, 0.14] 3.27
Kim et al., 2018 0.47 [0.32, 0.61] 2.60
Vukojevic et al., 2018 0.12 [0.10, 0.15] 3.26
Raffaeli et al., 2013 0.11 [0.08, 0.13] 3.27
Mhangara et al., 2020 0.08 [0.04, 0.13] 3.22
Heterogeneity: τ^2 = 0.01, I^2 = 97.21%, H^2 = 35.79 0.15 [0.08, 0.23]
Test of $\theta_i = \theta_j$: Q(8) = 51.15, p< 0.0001
Unexplained Pain
O'Donnell et al., 2013 (ProFess Trial) 0.11 [0.10, 0.11] 3.29
Westerlind et al., 2020 0.40 [0.34, 0.46] 3.16
Paolluci et al., 2016 0.30 [0.25, 0.34] 3.22
Kong et al., 2004 0.42 [0.33, 0.51] 2.98
Widar et al., 2002 0.15 [0.11, 0.19] 3.22
Indredavik et al., 2008 0.55 [0.49, 0.61] 3.14
Apperlos P,.2006 0.11 [0.07, 0.15] 3.23
Sacley et al., 2008 (Nothingham Stroke Register) 0.35 [0.27, 0.44] 3.03
Koivunen et al., 2015 0.51 [0.42, 0.59] 3.02
Naess et al., 2012 (The Bergen Stroke Registry) 0.49 [0.44, 0.55] 3.17
Jonson et al., 2006 (Lund Stroke Register) 0.31 [0.26, 0.37] 3.18
Hansen et al., 2012 0.46 [0.40, 0.52] 3.16
Ludstrom et al., 2009 0.21 [0.14, 0.27] 3.12
Paolluci et al., 2016 0.43 [0.33, 0.52] 2.99
Skaner et al., 0.37 [0.29, 0.45] 3.06
Katzan et al., 2018 0.44 [0.41, 0.47] 3.26
Heterogeneity: τ^2 = 0.02, I^2 = 98.07%, H^2 = 51.71 0.35 [0.28, 0.42]
Test of $\theta_i = \theta_j$: Q(15) = 1375.85, p<0.0001
Unexplained Sholder Pain
Gamble et al., 2012 0.42 [0.34, 0.51] 3.02
Lindgren et al., 2018 (Lund Stroke Register) 0.23 [0.18, 0.28] 3.20
Kuptniratsaikul et al., 2013 (TSRR registry) 0.16 [0.11, 0.21] 3.20
Heterogeneity: τ^2 = 0.02, I^2 = 94.84%, H^2 = 19.37 0.27 [0.12, 0.42]
Test of $\theta_i = \theta_j$: Q(2) = 25.65, p<0.0001
Overall 0.29 [0.23, 0.34] 100.00
Heterogeneity: τ^2 = 0.02, I^2 = 98.68%, H^2 = 75.85
Test of $\theta_i = \theta_j$: Q(31) = 1882.48, p<0.0001
Test of group differences: $Q_b(3)$ = 17.38, p<0.0001
0
20
40
60
80
100
Random-effects REML model

1.3 Bladder Dysfunction

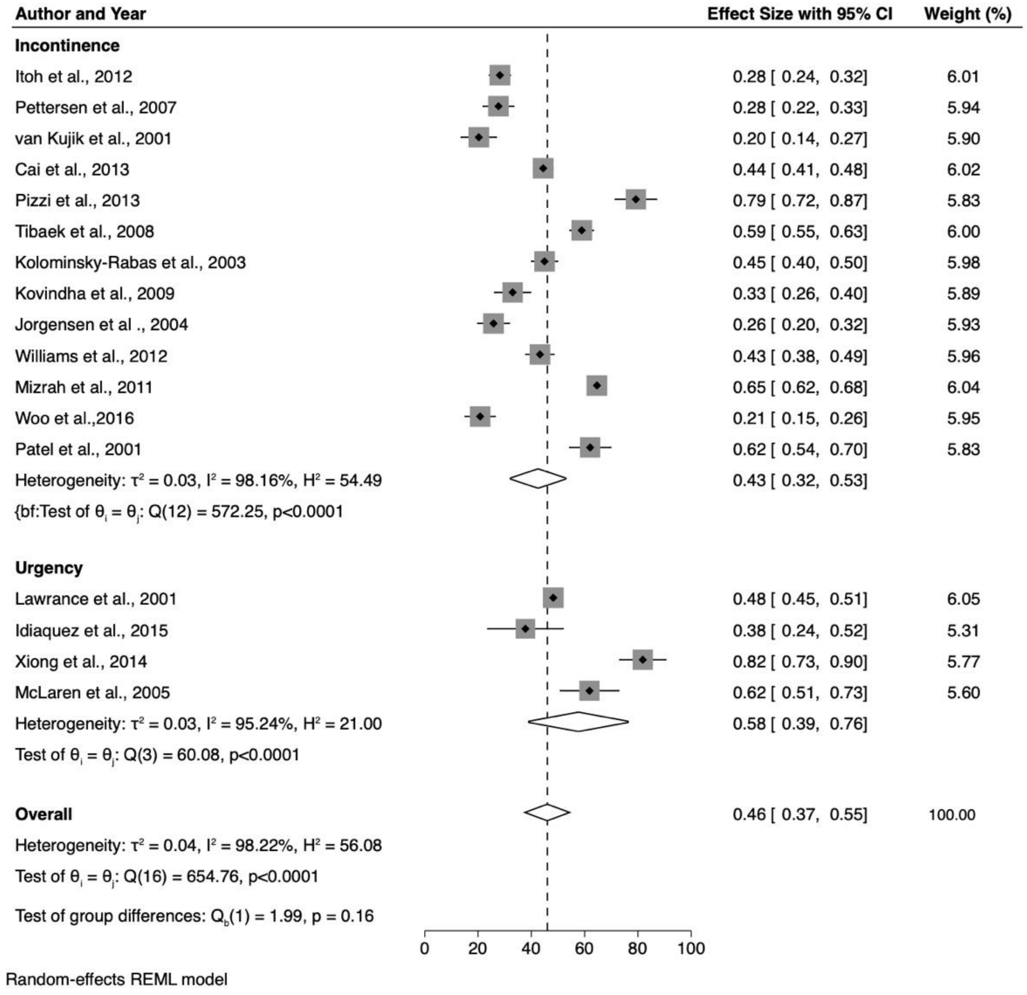

© 2025 Ozkan H et al. *JAMA Network Open*.

1.4 Sexual Dysfunction

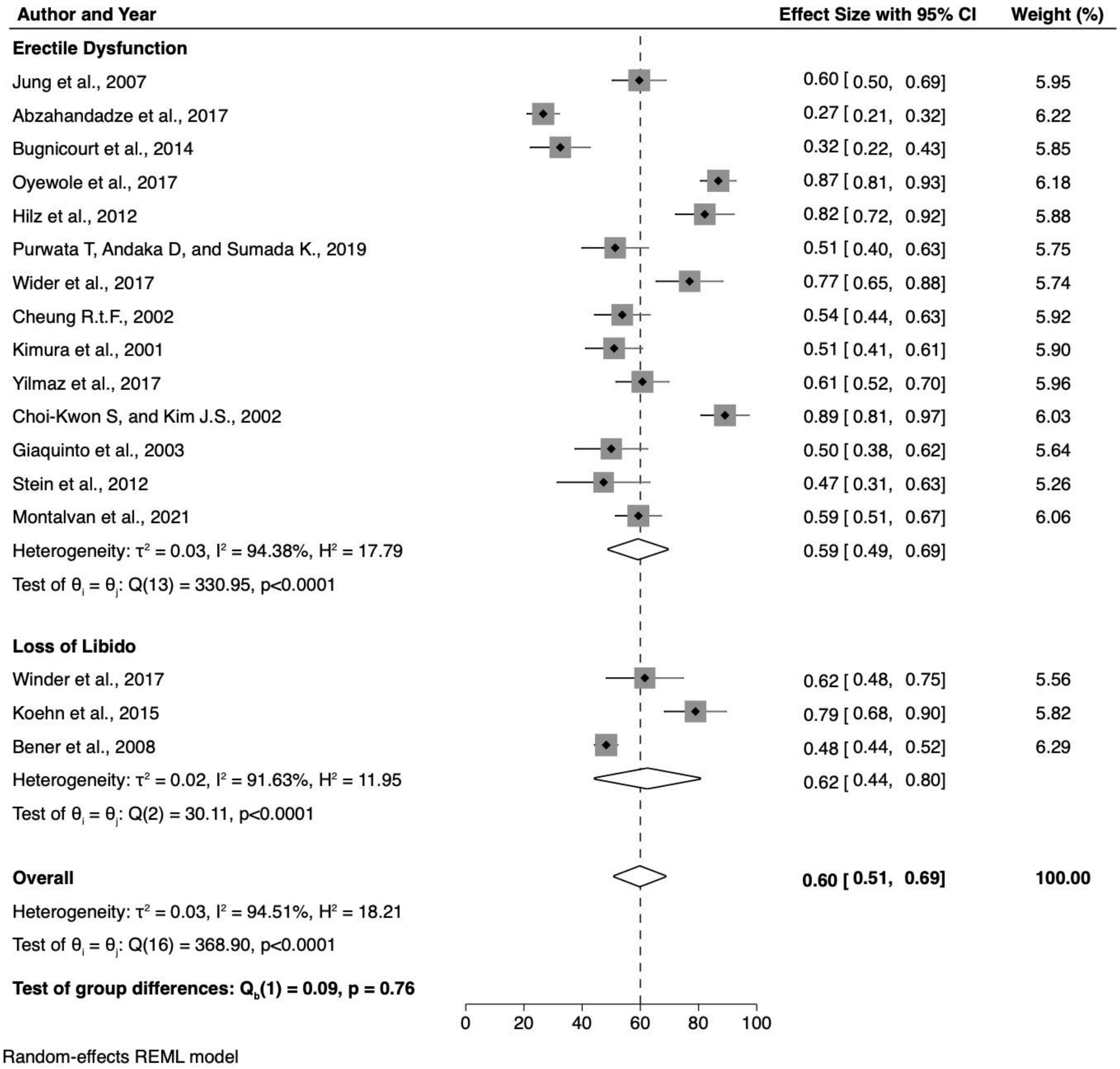

© 2025 Ozkan H et al. *JAMA Network Open*.

eTable 7. Subgroup analysis of study level characteristics

	Anxiety	Depression	Fatigue	Sleep Disturbance	Social Participation	Pain	Bladder Dysfunction	Constipation	Faecal Incontinence	Sexual Dysfunction
**Variable**	**Heterogeneity (I^2), P Value**									
**Age**										
≤ 55 years	95.69%, p< 0.001	93.56% p<0.001	94.12% p<0.001	96.65% p< 0.001	76.71% p= 0.04	99.59% p< 0.001	93.44% p< 0.001	**56.43% p= 0.013**	-	**49.38% p= 0.048**
≥ 56 years	94.29%, p< 0.001	97.80% p<0.001	95.68% p<0.001	94.40% p< 0.001	87.91% p= 0.41	97.39% p<0.001	98.32% p= 0.002	**37.14% p= 0.002**	98.16% p< 0.001	81.63% p< 0.001
**Sex**										
Female	94.06% p< 0.001	97.37% p<0.001	90.97% p< 0.001	94.40% p<0.001	**74.30% p< 0.001**	98.92% p< 0.001	96.36% p< 0.001	**60.92% p= 0.007**	78.42% p= 0.016	95.47% p< 0.001
Male	97.03% p< 0.001	97.40% p<0.001	96.70% p<0.001	93.83% p< 0.001	86.53% p= 0.069	97.38% p<0.001	98.38% p= 0.007	**36.37% p= 0.002**	98.78% p< 0.001	93.97% p< 0.001
**Stroke Type**										
Ischaemic	96.78% p< 0.001	96.15% p< 0.001	95.45% p< 0.001	94.53% p< 0.001	86.53% p<0.001	98.68% p< 0.001	96.64% p<0.001	**74.14% p= 0.001**	-	94.67% p< 0.001
Intracerebral Haemorrhage (ICH)	**10.71% p= 0.034**	**57.06% p= 0.002**	-	-	-	**15.32% p= 0.041**	-	**-**	-	-
Mixed Cohort	92.63% p<0.001	93.05% p< 0.001	94.52% p<0.001	76.83% p= 0.014	75.87% p< 0.001	97.88%	97.81% p< 0.001	**59.82% p= 0.029**	97.34% p< 0.001	92.25% p< 0.001

© 2025 Ozkan H et al. *JAMA Network Open*.

						p< 0.001				
**Outcome Measures**										
1	94.26% p<0.001	94.62% p< 0.001	93.72% p< 0.001	**72.67% p= 0.039**	88.04% p< 0.001	97.29% p< 0.001	98.82% p< 0.001	**52.51% p< 0.001**	-	85.39% p= 0.056
2	96.70% p<0.001	89.64% p< 0.001	94.32% p< 0.001	94.50% p< 0.001	81.53% p< 0.001	**67.81% p= 0.036**	96.41% p< 0.001	**42.17% p= 0.027**	-	**57.48% p< 0.001**
3	**52.99% p= 0.009**	93.54% p< 0.001	89.00% p< 0.001	92.84% p< 0.001	-	98.23% p< 0.001	-	-	-	95.34% p< 0.001
4	-	99.03% p< 0.001	96.24% p< 0.001	-	86.51% p= 0.066	98.77% p< 0.001	97.69% p< 0.001	**37.67% p= 0.008**	98.74% p< 0.001	-
**Time to follow-up (month)**										
1 to 3	93.38% p< 0.001	94.31% p< 0.001	91.63% p< 0.001	97.67% P<0.001	91.56% p<0.001	98.94% p<0.001	96.36% P<0.001	-	-	-
6 to 12	85.6% p<0.001	94.83% p< 0.001	94.04% P< 0.001	90.37% P<0.001	**48.12% p= 0.008**	96.22% p<0.001	97.27% p<0.001	**50.2% p= 0.011**	-	**36.4% P= 0.012**
13 to 24	-	-	91.02% p<0.001	83.35% p <0.001	-	96.61% p<0.001	-	-	-	-
25 to 36	94.94% p<0.001	87.92% p<0.001	-	-	**36.51% p= 0.004**	-	-	-	-	86.92% P<0.001
>37	91.23% p<0.001	89.13% P<0.001	98.55% p <0.001	96.53% P<0.001	-	98.35% p<0.001	97.56% p<0.001	-	-	-

© 2025 Ozkan H et al. *JAMA Network Open*.

Outcome Measure Anxiety: 1= HADS (Hospital Anxiety and Depression Scale); 2= Other Patient Reported Outcome Measures (PROMs); 3= Interview. Outcome Measure Depression: 1= HADS (Hospital Anxiety and Depression Scale); 2= BDI (Beck's Depression Inventory); 3= GAD-7 (Generalised Anxiety Disorder-7 item scale); 4= Other Patient Reported Outcome Measures (PROMs). Outcome Measure Fatigue: 1= FAS (Fatigue Severity Scale); 2= Interview; 3= Checklist individual strength questioner; 4= Interview. Outcome Measure Sleep Disturbance: 1= ESS (Epworth Sleepiness Score); 2= Polysomnography; 3= BSQ (Berlin Sleep Questioner); 4= blank. Outcome Measure Social Participation: 1= SSQT (Social Support Questionnaire – Transaction); 2= NewSQL (Newcastle Stroke-Specific QoL); 3= - (blank); 4= Other Patient Reported Outcome Measures (PROMs). Outcome Measure Pain: 1= SF-36 (Short Form (36) Health Survey); 2= VAS (Visual Analogue Scale); 3= PROMIS (The Patient Reported Outcomes Measurement Information System); 4= Other Patient Reported Outcome Measures (PROMs). Outcome Measure Bladder Dysfunction: 1= BI (Barthel Index); 2= Modified Barthel ADL Index; 3= blank; 4= Interview. Outcome Measure Constipation: 1= BI (Barthel Index); 2= Modified Barthel ADL Index; 3= blank; 4= Interview using Other Patient Reported Outcome Measures (PROMs). Outcome Measure Faecal Incontinence: 1= blank; 2= blank; 3=blank; and 4= Interview.

© 2025 Ozkan H et al. *JAMA Network Open*.
