# Supplementary material for: Prevalence, Trajectory, and Factors Associated With Patient-Reported Nonmotor Outcomes After Stroke: A Systematic Review and Meta-Analysis
Source: JAMA Netw Open. 2025 Feb 21;8(2):e2457447. doi: 10.1001/jamanetworkopen.2024.57447 (PMC11846016; doi:10.1001/jamanetworkopen.2024.57447)
Supplement: Supplement 2. — Data Sharing Statement [file jamanetwopen-e2457447-s002.pdf]

Data Sharing Statement

Ozkan. Prevalence, Natural History, and Factors Associated With Patient-Reported Nonmotor Outcomes After Stroke. *JAMA Netw Open*. Published February 14, 2025.
doi:10.1001/jamanetworkopen.2024.57447

Data
Data available: Yes
Data types: Data dictionary
How to access data: Raw data for the meta-analysis
When available: With publication

Supporting Documents
Document types: Statistical/analytic code
How to access documents: h.capar@ucl.ac.uk
When available: With publication

Additional Information
Who can access the data: Researchers whose proposed use of the data has been approved
Types of analyses: for a specified purpose
Mechanisms of data availability: After approval of a proposal, and with a signed data access agreement
